# Prenatal Stress Increases the Risk of the FPR2-related Dysfunction in the Brain's Resolution of Inflammation: A Study on the Humanized APP^NL-F/NL-F^ Mouse Model of Alzheimer's Disease

**DOI:** 10.2174/011570159X345385241004060055

**Published:** 2025-03-12

**Authors:** Ewa Trojan, Jakub Frydrych, Władysław Lasoń, Agnieszka Basta-Kaim

**Affiliations:** 1 Laboratory of Immunoendocrinology, Department of Experimental Neuroendocrinology, Maj Institute of Pharmacology, Polish Academy of Sciences, 12 Smętna St. 31-343 Kraków, Poland

**Keywords:** Prenatal stress, aging, knock-in mice (APP^NL-F/NL-F^), formyl peptide receptor 2, resolution of inflammation, NFκB-A20 pathway, inflammasome NLRP3, cellular senescence

## Abstract

**Introduction:**

Brain aging is a complex process involving genetic, neurodevelopmental, and environmental factors. Inherent features of this process are cellular senescence, the development of senescence-associated secretory phenotype (SASP), and prolonged inflammation.

**Methods:**

Recently, progress has been made in understanding the biological roles of FPR2 receptors and their ligands in the mechanism of inflammation resolution (RoI) in the brain. However, the number of studies comparing the influence of prenatal stress (PS) on RoI in physiological aging and neurodegenerative disorders pathology is very limited, and the data need to be more consistent. Here, we examined whether PS can condition the pattern of age-dependent cognitive and RoI changes in the prefrontal cortex and hippocampus in wild-type and hAPP^NL-F/NL-F^ KI male mice.

**Results:**

We discovered that in aging, the memory deficits are accompanied by the limitation of the availability of pro-resolving FPR2 ligands, the rising proinflammatory microglia polarization, and inflammatory ligands mediated FPR2 overactivation. Moreover, the present study suggested the subtle role of the RoI deficits in creating brain cells' senescence and shifting the immunomodulators to the proinflammatory direction. PS has been revealed as a substantial factor modulating the profile of inflame-aging in a manner strongly determined by the age of animals and the brain structure under study, mainly in hAPP^NL-F/NL-F^ KI male mice.

**Conclusion:**

Our results identify the FPR2 receptors as a driver regulating the RoI process in the brain and highlight that PS has diversified the picture of age-dependent neurodegenerative pathology.

## 1 INTRODUCTION

Alzheimer's disease (AD) is an age-related progressive neurodegenerative disease, the pathological mechanism of which has not been fully elucidated. Alzheimer's disease is characterized by two main features: the accumulation of tangled, hyperphosphorylated tau proteins inside neurons, forming neurofibrillary tangles (NFTs), and the buildup of amyloid beta (Aβ) protein outside neurons, forming plaques in the brain [1-3]. Nevertheless, these crucial neuropathological features are not always present in the course of AD. At the same time, their presence in the brain does not unambiguously determine the development of AD. Currently, the prevailing view is that the pathology of AD is determined by various factors that coexist, creating an age-dependent image of neurodegeneration. Among them are neuronal loss, synaptic dysfunction, brain atrophy, and changes in the brain inflammatory response, which involves numerous molecular, cellular, and physiological reactions responsible for the initiation, execution, and resolution of inflammation (RoI).

Previous studies have indicated that the regulation of the inflammatory process is complex. The onset-to-peak phase of the response to injury/infection is followed by temporally appropriate and controlled resolution, leading to homeostasis and normal function [4]. The different phases of response do not occur sequentially but overlap [5]. Dysfunction of RoI in the brain causes uncontrolled chronic inflammation associated with the aberrant activation of the main immunocompetent cells in the brain, called microglia, and elevated production of various proinflammatory and harmful factors [6]. Moreover, disturbed RoI modulates the microglial phenotype, as indicated not only by a shift in the proinflammatory direction but also by a loss of proper function and senescence [7].

RoI is regulated by specialized pro-resolving mediators (SPMs), which include lipoxin A4 (LXA4) and annexin 1 (ANXA1) [8]. Specialized pro-resolving mediators (SPMs) exhibit dual functions by both reducing inflammation and promoting resolution [9]. They help mitigate oxidative stress by limiting the production of reactive oxygen and nitrogen species, as well as enhancing the activity of natural antioxidant systems [10]. SPMs exert their biological effects through interactions with various specific membrane receptors. Among them formyl peptide receptors, with formyl peptide receptor 2 (FPR2) being particularly intriguing [11]. FPR2 is a highly versatile receptor capable of interacting with a wide range of ligands, activating different signal transduction pathways depending on factors such as ligand structure, concentration, and cell type involved. FPR2, a “promiscuous” receptor, can mediate anti-inflammatory effects when activated by lipoxin A4 (LXA4), which is an anti-inflammatory lipid and the first endogenous FPR2 ligand to be identified, or Annexin A1, a glucocorticoid-regulated protein that is involved in the adhesion mechanisms of leukocytes [12]. In contrast, this receptor mediates proinflammatory effects if activated by the amyloidogenic peptide serum amyloid A, β-amyloid, or prion protein PrP (106-126), which are associated with chronic neuroinflammation and amyloidosis. The ability of FPR2 to mediate contrasting effects is mechanistically related to the agonist-biased and receptor-conformational changes that can be induced in a ligand-specific manner [13]. Thus, a wide range of responses are mediated by FPR2 expressed in the brain, mainly in microglia and astrocytes and, to a lesser extent, in neuronal cells, suggesting that this receptor may represent a homoeostatic target for reducing neuroinflammation.

Although age is considered the main causative factor of AD pathology, early adverse life experiences may also be relevant to the development of AD [14, 15]. Prenatal maternal stress (PS) is associated with physiological, neurological, and psychological consequences in offspring [16, 17]. Several potential and nonexclusive mechanisms by which PS induces adverse neonatal/offspring outcomes have been suggested, including epigenetic alterations [18] and dysregulation of the maternal/fetal HPA axis [19]. Previous descriptive studies have also suggested that maternal stress impacts neonatal immunity [19] and/or contributes to an increased risk of immune-related disorders in children [20-22]. The deleterious consequences of PS not only affect the first generation of newborns but also may be transmitted across subsequent generations [23, 24] because when the brain is still developing, the effect of PS has more pronounced and longer-lasting detrimental effects compared to that in adults [25]. Stressful events during pregnancy potentiate the release of glucocorticoid hormones from maternal adrenal glands and proinflammatory mediators [26, 27]. These substances can cross the placental barrier *via* the bloodstream, affecting the development of the fetal brain [28], particularly impacting regions like the frontal cortex, amygdala, and hippocampus [14, 29, 30]. Furthermore, the effects of prenatal stress (PS) on the brain result in enduring alterations in offspring behavior [31-33], often leading to impairments in spatial learning and memory functions [34, 35]. Thus, the potential mechanisms through which PS exerts its effects are robust and can change over time. However, the potential impact of age on the PS-induced effects on RoI regulation and the pathogenesis of AD is poorly understood.

Considering the abovementioned data, in the present study, we assessed the age-dependent impact of PS on the regulation of inflammation resolution in selected brain areas in both physiological (WT mice) and pathological (aging processes related to the development of AD pathology) conditions. We employed a mouse model with the human amyloid precursor protein (hAPP) inserted (knocked-in) into the mouse genome [3, 36, 37] by incorporating both Swedish and Beyreuther/Iberian mutations. Based on previously published results, hAPP^NL-F^ (KI) mice integrate many aspects of human late-onset AD (LOAD) [37, 38]. One of the hallmarks of AD is the aggregation and accumulation of extracellular amyloid β (Aβ) in the brain. To replicate these Aβ pathologies and contribute to a better understanding of AD, preclinical mouse models have been created. Notably, transgenic mice expressing the amyloid precursor protein gene (App) with disease-associated mutations (*e.g*., APP/PS1) have played a pivotal role in elucidating disease mechanisms and exploring therapeutic candidates. However, it is crucial to acknowledge that transgenic models have limitations, including overexpression-related artifacts, unintended gene disruption, and variable expression patterns, which can diminish their relevance as pre-clinical AD models.

To overcome these limitations, the development of human APP-knock-in (KI) mice represents a significant advancement. APP-KI mice having humanized mouse Aβ sequences with three familial AD mutations (Swedish, Iberian, and Arctic), were introduced through a KI strategy. This approach may mitigate the shortcomings associated with transgenic models. In the present study, as previously [38], we used the APP^NL-F/NL-F^ model (the Swedish mutation, NL, and the Beyreuther/Iberian mutation, F), which exhibits a markedly increased human Aβ42 and Aβ40 ratio starting at 2 months of age, with plaques evident after 12 months of age. Moreover, cognitive deficits develop at the age of 15-16 months [36, 38]. Thus, this model offers a more relevant representation of amyloid pathology, cognitive deficits, and disease progression seen in human Alzheimer's disease, known as late-onset AD pathology (LOAD). This makes it a valuable tool for studying the underlying mechanisms of AD and for evaluating potential therapeutic interventions.

Since the molecular underpinnings of PS-induced age-dependent RoI dysfunction, cognitive deficits, and neurodegenerative changes have been only partially elucidated, we used various tests to assess age-dependent alterations in spatial learning, memory ability, and cognitive functions. In biochemical studies, we explored age-dependent changes in the Aβ42/Aβ40 ratio in the hippocampi and frontal cortices of adult offspring. Moreover, the age-dependent impact of PS on the expression of FPR2 and its pro-resolving ligand (LXA4, ANXA1) in both brain areas was evaluated. Since the balance of chemically and functionally diverse FPR2 ligands determines the effectiveness of RoI, we evaluated the mRNA expression of pro-inflammatory and anti-inflammatory microglia and some senescence markers (*iNos*, *Cd40*, *Ym1*, *Arg1*, p53, p16, and pRb), as well as the levels of selected cytokines (IL-1β, IL-10, and TGF-β), which indicate the functional phenotype of these cells and are crucial for obtaining a holistic view of RoI.

Finally, to shed more light on the molecular background of the impact of early adverse manipulation on the crucial downstream signaling pathways related to the FPR2-mediated RoI in the selected brain areas, the levels of phosphorylation of NF-κB and A20 (also known as tumor necrosis factor alpha-induced protein 3; TNFAIP3) as well as the NLRP3 inflammasome components (caspase-1, NLRP3, ASC) were investigated.

## 2 MATERIALS AND METHODS

### 2.1 Animals

The research was conducted using C57BL/6 mice sourced from Charles River (Sulzfeld, Germany. The APP^NL-F/NL-F^ mice (knock-in; KI) were sourced from the Riken Institute's breeding colony (Riken Brain Science Institute in Japan, Laboratory for Proteolytic Neuroscience, overseen by Dr. Takaomi Saido). All mice were kept under standard conditions, including a room temperature of 23°C, a 12-hour light/12-hour dark cycle with lights on at 06:00 am, and unrestricted access to both food and water. The methodology for breeding and genotyping was described previously [38]. In the experiments, two types of mice were used: wild-type homozygous (WT/WT) and transgenic homozygous (APP^NL-F/NL-F^) mice. These mice were divided into two groups, one containing control mice (unstressed) and the other containing mice subjected to a prenatal stress procedure (PS). All the experimental procedures strictly adhered to the guidelines established by the Committee for Laboratory Animal Welfare and Ethics of the Maj Institute of Pharmacology, Polish Academy of Sciences, located in Cracow, Poland, and were carried out under approval No. 258/2018 and 223/2023.

### 2.2 Stress Procedure

The PS procedure, as detailed in the study by Trojan *et al*. [38], involved exposing half of the pregnant females to daily stress sessions lasting 45 minutes at unpredictable times of the day during the final week of gestation, spanning from E12.5 to E18.5. These sessions consisted of placing the pregnant mice inside plastic cylinders and subjecting them to restraint stress. Following each 45-minute restraint session, the stressed pregnant mice (KI stressed -n = 10; WT stressed -n = 10) were returned to their home cages. In contrast, the control pregnant females (KI control -n = 10; WT control -n = 10) were not subjected to any stressors and were left undisturbed during this period. In our *in vivo* experiments, we selected male offspring from litters once they reached 21 days old. These male mice were then housed in groups of five per cage and maintained under typical living conditions. Behavioral assessments were conducted at the ages indicated in each figure legend. Following the completion of behavioral tests, mice were sacrificed one week later, and their brains were harvested for subsequent analyses.

### 2.3 Morris Water Maze Test

To assess memory function, the standard Morris water maze test was performed as described in our previous study [38]. In this test, mice were trained to locate a hidden platform submerged approximately 1 cm below the water's surface in a circular pool filled with water at a temperature of approximately 23°C. The inner walls of the pool were white, and the platform was transparent. To aid analysis, computer software was used to divide the pool into four quadrants: northeast (NE), northwest (NW), southeast (SE), and southwest (SW). The escape platform was consistently positioned in the northeast quadrant (NE) throughout the training phase, which lasted five consecutive days. On testing Days 1 to 5, the mice underwent four training trials each day. During these trials, they were placed sequentially into each of the four quadrants in a random order. If a mouse failed to locate the platform within 60 seconds, it was gently placed on the platform. The mouse remained on the platform for 20 seconds before being removed from the maze, dried with a towel, and returned to its home cage. The distance needed to swim to find the submerged platform during these trials was recorded. The probe trials were performed without a platform for one minute to assess memory retention on Day 6. The distance traveled in the NE zone, and the latency to first enter the platform zone were noted. Both the training and the test data were recorded by a video camera and evaluated with ANYMaze video-tracking software (UgoBasile, Italy).

### 2.4 Open Field Test

The open field (OF) test was performed as we described previously [39]. The animals were tested in a square box (40 × 40 × 40 cm^3^) illuminated in the central region. The light intensity was approximately 120 lux in the central area (20 × 20 cm^2^) and approximately 50 lux outside the central area. Each session lasted 30 min. The distance moved (m), mobility (%), and velocity (cm/s) were measured using video tracking software EthoVision XT 15 (Noldus, Wageningen, the Netherlands).

### 2.5 Tissue Sample Collection

Mice were sacrificed by rapid decapitation at 12, 15, and 18 months of age. The frontal cortex (Cx) and hippocampus (Hp) were carefully isolated, and the tissues were promptly frozen using dry ice and stored at -80°C. The tissue preparation and determination of protein concentration were carried out following the methodology described earlier by us [40]. The lysates of the samples were preserved at -80°C until they were utilized for enzyme-linked immunosorbent assays (ELISAs).

### 2.6 Enzyme-linked Immunosorbent Assays (ELISAs)

The levels of mouse Interleukin 1 Beta (IL-1β), Interleukin 10 (IL-10), Transforming Growth Factor β1 (TGF-β1), Annexin A1 (ANXA1), Formyl Peptide receptor 2 (FPR2), The retinoblastoma protein (pRb), Cyclin Dependent Kinase Inhibitor 2A (CDKN2A), Tumor Protein 53 (TP53), Nuclear Factor-κB p65 (NF-κB) (all eight from FineTest, Wuhan, China), phospho-Nuclear Factor-κB p65 (p-NF-κB) (from Invitrogen, Waltham, Massachusetts, USA) Tumor Necrosis Factor Alpha Induced Protein 3 (TNFaIP3), PYD and CARD Domain Containing Protein (PYCARD), Caspase 1 (CASP1), NLR Family, Pyrin Domain Containing Protein 3 (NLRP3) (all four from ELK Biotechnology, Wuhan, China), Lipoxin A4 (LXA4) (from Cusabio, Houston, TX, USA), and Human Amyloid β (Aβ; aa1-40 and aa1-42) (both from R&D Systems, Minneapolis, MN, USA) in Cx and Hp samples were quantified by ELISA kits obtained from commercial sources, and the procedures outlined by the manufacturers were strictly adhered to for analysis. Absorbance measurements were conducted using a Tecan Infinite M200 Pro (TECAN, Männedorf, Switzerland) set up to the required wavelength.

The minimum detectable concentrations of each protein were as follows: IL-1β, 7.5 pg/ml; IL-10, 9.375 pg/ml; TGF-β1, 18.75 pg/ml; ANXA1, 0.094 ng/ml; FPR2, 18.75 pg/ml; pRb, 0.938 ng/ml; CDKN2A, 23.438 pg/ml; TP53, 37.5 pg/ml; NF-κB, 0.188 ng/ml; p-NF-κB, not applicable; TNFaIP3, 0.112 ng/ml; PYCARD, 3 pg/ml; CASP1, 13.5 pg/ml; NLRP3, 0.053 ng/ml; LXA4, 15.6 pg/ml; Aβaa1-40, 3.97 pg/ml; and Aβaa1-42, 2.33 pg/ml. The precision of both intra- and inter-assay measurements varied depending on the specific attributes of the assay.

### 2.7 Isolation of RNA, Conversion to Complementary DNA (cDNA), and Quantitative Real-time Polymerase Chain Reaction (qPCR)

Total RNA was isolated from the Cx and Hp following the protocol provided by the Total RNA Mini Kit from A&A Biotechnology (Gdynia, Poland). NG dART RT Kit (EURx, Gdansk, Poland) was used for cDNA synthesis. Gene expression levels were quantified using TaqMan primers and probes (*iNOS* (Mm00440502_m1), *Cd40* (Mm00441891_m1), *Ym1* (Mm00657889_mH), and *Arg1* (Mm00475988_m1)) with the FastStart Universal Probe Master (Rox) Kit (Roche, Basel, Switzerland) using the CFX96 Real-Time System (Bio-Rad, Hercules, CA, USA) as described previously [40, 41]. The threshold value (Ct) for individual samples was established in the exponential phase of PCR, and the ΔΔCt method was used for data analysis. *Gapdh* (Mm99999915_g1) was used as the reference gene.

### 2.8 Statistical Analysis

All of the statistical analyses were performed using Statistica software, version 10.0 (StatSoft, Tulsa, USA). The outcomes of the behavioral studies are presented as the mean ± SD. Levene’s test was used for homogeneity of variance assessment. The normality of variable distribution was assessed by the Shapiro-Wilk test. The differences between means were analyzed using multifactorial analysis of variance (ANOVA) with Duncan’s post hoc test when appropriate. A value of *p* < 0.05 was considered to indicate statistical significance. All of the data are presented as mean ± SD. n = 3-8 (behavioral studies); n = 3-4 (biochemical analyses). Graphs were generated using GraphPad Prism 7. Detailed findings regarding the effects of genotype (GEN), prenatal treatment (PS), age (Y), and their interactions can be found in the Supplementary Tables provided.

## 3 RESULTS

### 3.1 Morris Water Maze (MWM) Test

In line with our previous observations [38], in the Morris Water Maze test, no disparities were observed in the total distance traveled (in meters) to locate the concealed platform across all groups during the 5-day learning trials at ages 9, 12, or 15 months. This suggests that all mice exhibited normal motor and visual capabilities to complete the task. (Table **1**, Table **S1**). Regarding the differences between 18-month-old KI and WT mice, a significant increase in the time needed to find the platform was observed but only at *Day 5 (*p* = 0.000489). Importantly, parental stress (PS) prolonged the time needed to find the platform in both WT (^Day 2: *p* = 0.022302) and KI mice (#Day 2: *p* = 0.010554; #Day 4: *p* = 0.000062; #Day 5: *p* = 0.002273). What’s more, the effect of PS on KI mice was significantly greater than that on WT mice ($Day 4: *p* = 0.000131; $Day 5: *p* = 0.000073) (Table **1**; Table **S1**).

In the probe trial, after removing the platform, the distance traveled in the NE zone, and the latency to the first entry to the platform zone was recorded. At 9 and 12 months of age, we did not observe an impact of the knock-in procedure on any of the analyzed parameters (Table **1**). KI mice traveled a shorter distance in the NE zone at the ages of 15 (*p* = 0.006897) and 18 months (*p* = 0.000568). Additionally, the latency to reach the platform quadrant was greater in the 18-month-old KI mice than in the WT mice (*p* = 0.000654) (Table **1**; Table **S1**).

PS led to a decrease in the distance traveled in the quadrant where the platform was located by the 18-month-old WT mice (*p* = 0.004591; Table **1**). PS also shortened the time spent in the NE zone in KI animals *vs.* WT mice subjected to the stress procedure during the perinatal period (15-month-old: *p* = 0.008811; 18-month-old: *p* = 0.008594). PS also increased the latency to the first entry to the platform zone in both the WT (*p* = 0.034373) and KI (*p* = 0.006129) mice, but the effect of PS on the KI mice was significantly greater than that on the WT mice (*p* = 0.000291) (Table **1**; Table **S1**).

These results suggest that spatial learning and memory abilities in the KI group deteriorated with age. Additionally, the prenatal stress procedure exacerbated the cognitive impairments observed in the Morris Water Maze test.

### 3.2 Open Field Test (OF)

To test the age-dependent effects of the mutation on exploratory behavior, we performed an open-field test. First, the animals were introduced to an empty open field with illumination in the central area. There was a difference between 12-month-old WT and KI animals in terms of the distance moved (*p* = 0.037160; Table **2**) and velocity (*p* = 0.038577; (Table **2**, Table **S2**).

In general, an age-dependent decrease in exploratory behavior was observed. Control WT and KI mice aged 15 and 18 months were characterized by a reduced distance traveled (15-month-old WT: *p* = 0.010904; 18-month-old WT: *p* = 0.001832; 18-month-old KI: *p* = 0.002803; Table **2**), a decreased velocity (15-month-old WT: *p* = 0.011301; 18-month-old WT: *p* = 0.001105; 18-month-old KI: *p* = 0.004141) and decreased mobility (15-month-old WT: *p* = 0.013721; 18-month-old WT; *p* = 0.002072; 18-month-old KI: *p* = 0.006449) compared to those of 9-month-old mice with the same genotype (Table **2**; Table **S2**).

In addition to age, PS also affected behavioral parameters. The OF test showed that in comparison to those of 9-month-old animals, both WT and KI mice had shorter distances (12-month-old WT: *p* = 0.000286; 15-month-old WT: *p* = 0.009540; 18-month-old WT: *p* = 0.007459; 18-month-old KI: *p* = 0.027402; Table **2**); and lower velocities (12-month-old WT: *p* = 0.000337; 15-month-old WT: *p* = 0.009975; 18-month-old WT: *p* = 0.001387; 18-month-old KI: *p* = 0.027595). Decreased mobility was observed only in 18-month-old KI mice (*p* = 0.027123) (Table **2**; Table **S2**). In summary, we observed age-dependent changes in locomotor activity. On the other hand, the knock-in procedure and stress during the perinatal period only slightly affected locomotor activity in the present study.

### 3.3 Age-dependent Impact of PS on FPR2 Level and Its Ligands: AnxA1, LXA4, and Aβ42/Aβ40 in the Hippocampi and Frontal Cortices of Normal Aging (WT) and Knock-in APP^NL-F/NL-F^ Mice

Formyl peptide receptor 2 (FPR2) is a high-affinity binding partner of Aβ_1-42_. A large body of evidence suggests that FPR2 serves as a receptor mediating Aβ_1-42_-elicited proinflammatory responses. However, FPR2 has also been found to mediate anti-inflammatory and exert pro-resolving effects when activated by Annexin A1 (AnxA1) and by the specialized pro-resolving mediator lipoxin A4 (LXA4). The balance between these two effects is responsible for the effective resolution of inflammation (RoI). Therefore, in the next set of experiments, we estimated the impact of age and PS on the protein levels of FPR2, AnxA1, LXA4 and on Aβ42/Aβ40 ratio in the frontal cortex and hippocampus samples from 12-, 15-, and 18-month-old WT and KI mice.

All biochemical and molecular studies were conducted in animals aged 12, 15, or 18 months.

**Fig. (1):**
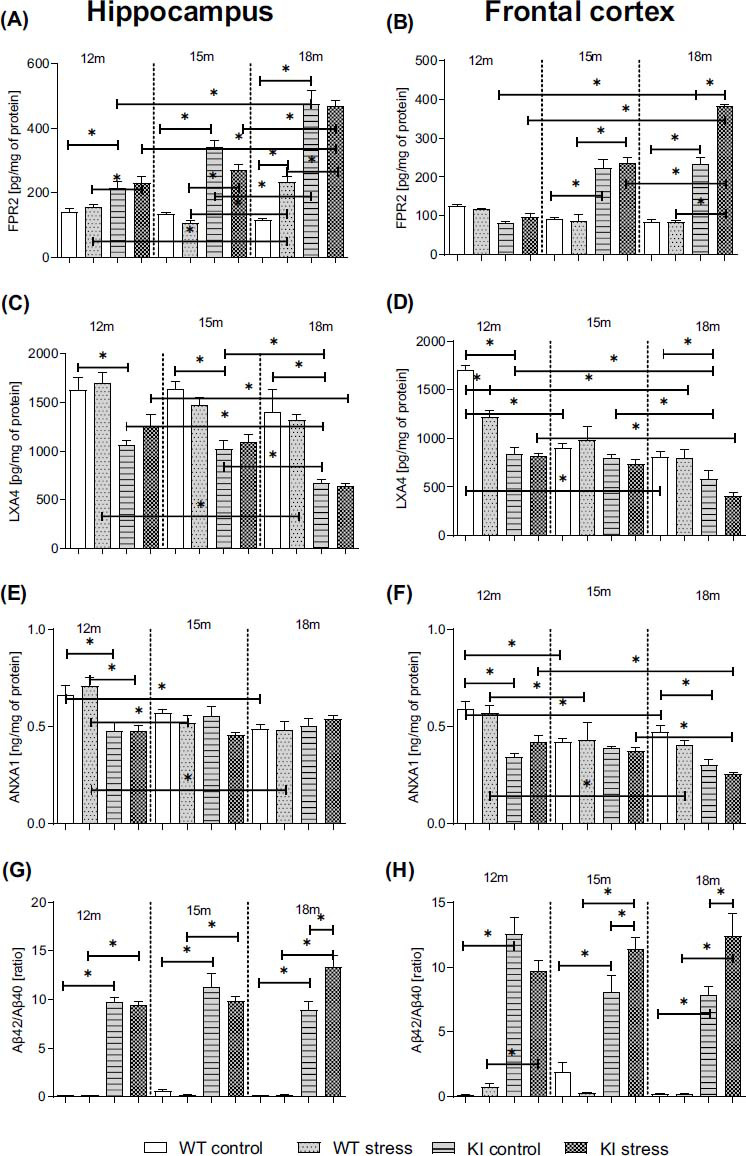
Age-dependent impact of prenatal stress on formyl peptide receptor 2 (FPR2; pg/mg of protein; **A**, **B**), lipoxin A4 (LXA4; pg/mg of protein; **C**, **D**), annexin A1 (ANXA1; ng/mg of protein; **E**, **F**) levels and the amyloid-β Aβ42/Aβ40 ratio (Aβ; ratio; **G**, **H**) in the hippocampus and frontal cortex of 12-, 15-, and 18-month-old wild-type (WT) and APP^NL-F/NL-F^ (KI) mice. ANOVA with Duncan’s *post hoc* test (*p < 0.05, n = 3-4 mice in each group, error bars are standard deviations).

Regarding the differences between WT and KI mice, significant increases in FPR2 protein levels were observed in both analyzed brain areas of control mice at the ages of 12 (Hp: *p* = 0.014870), 15 (Hp: *p* = 0.000017; Cx: *p* < 0.0001), and 18 months (Hp: *p* = 0.000019; Cx: *p* < 0.0001) (Figs. **1A**, **B**) (Table **S5**) and in those of prenatally stressed KI mice aged 12 (Hp: *p* = 0.010450), 15 (Hp: *p* = 0.000019; Cx: *p* = 0.00020), and 18 months (Hp: *p* = 0.000053; Cx: *p* = 0.000018) compared to age-matched WT PS mice. The FPR2 level observed in the 18-month-old KI mice was significantly greater than that observed in the 12- (Hp: *p* = 0.000022; Cx: *p* = 0.000020) and 15- (Hp: *p* = 0.03775) month-old KI mice (Table **S5**).

After the PS procedure, we also observed an age-dependent increase in FPR2 levels in 18-month-old mice compared with 15-month-old (Hp: KI: *p* = 0.000063; WT: *p* = 0.000123; Cx: KI: *p* = 0.000134) and 12-month-old (Hp: KI: *p* = 0.000031; WT: *p* = 0.000028; Cx: KI: *p* = 0.000018) mice and 18-month-old genotype matched mice. Moreover, the effect of PS was significantly greater in the frontal cortex of 18-month-old KI mice (*p* = 0.000062) and in the hippocampus of 18-month-old WT animals (*p* = 0.000275) than in age- and genotype-matched mice (Figs. **1A**, **B**) (Table **S5**).

Analyses of brain tissue samples revealed a lower level of LXA4 in the hippocampus of 12-month-old KI mice (*p* = 0.001047), 15-month-old (*p* = 0.000388), and 18-month-old (*p* = 0.000047) and the frontal cortex of 12-month-old (*p* = 0.000033) and 18-month-old control transgenic animals (*p* = 0.043392) than in WT mice of the same age (Figs. **1C**, **D**) (Table **S5**).

Moreover, 18-month-old mice were characterized by a lower level of LXA4 than 12-month-old (KI: Hp *p* = 0.010176; Cx *p* = 0.026108; WT: Cx *p* = 0.000023) or 15-month-old (KI: Hp *p* = 0.010176) mice were compared with genotype-matched mice (Figs. **1C**, **D**). Additionally, the level of LXA4 in the frontal cortex was lower in 15-month-old WT mice (*p* = 0.000055) than in 12-month-old WT animals (Fig. **1D**).

Prenatal stress led to a decrease in LXA4 levels in the frontal cortex of 12-month-old (*p* = 0.000163) WT mice. Importantly, in 18-month-old WT and KI mice, PS decreased the LXA4 level compared to that in 12- (Hp:KI: *p* = 0.000248; WT: *p* = 0.021412; Cx:KI: *p* = 0.000809; WT: *p* = 0.000549) and 15- (Hp:KI: *p* = 0.005023; Cx:KI: *p* = 0.003728) genotype-matched mice (Figs. **1C**, **D**) (Table **S5**).

Our results demonstrated that the level of ANXA1 was lower in both brain areas of 12-month-old KI mice (Hp: *p* = 0.001642; Cx: *p* = 0.000033) and in the frontal cortex of KI animals at the age of 18 months (*p* = 0.002056). Moreover, compared to 12-month-old genotype-matched animals, the oldest group of WT mice exhibited a lower level of ANXA1 (Hp: *p* = 0.002463; Cx: *p* = 0.017492). Similar results were obtained for the frontal cortex of 15-month-old mice (*p* = 0.001475) (Figs. **1E**, **F**) (Table **S5**).

The influence of stress during pregnancy on ANXA1 levels in the hippocampus was greater in 12-month-old KI mice (*p* = 0.000157) than in WT mice.

Furthermore, statistical analysis of the present study revealed an age-dependent decrease in ANXA1 levels in the hippocampus and frontal cortex of 15- (Hp: *p* = 0.001202; Cx: *p* = 0.005866) and 18- (Hp: *p* = 0.000217; Cx: *p* = 0.002525) WT mice subjected to a prenatal stress procedure compared to those in 12-month-old mice. In KI mice, a decreased level of ANXA1 was observed only in the frontal cortex of 18-month-old animals subjected to prenatal stress (*vs.* 12-month-old: *p* = 0.002241; *vs.* 15-month-old: *p* = 0.017384) (Figs. **1E**, **F**) (Table **S5**).

Analyses of brain tissue samples revealed that KI mice had increased hippocampal and cortical Aβ42/Aβ40 ratio (12-month-old: Hp: *p* < 0.0001; Cx: *p* < 0.0001; 15-month-old: Hp: *p* < 0.0001; Cx: *p* < 0.0001; 18-month-old: Hp: *p* < 0.0001; Cx: *p* < 0.0001) compared to that in age-matched WT control mice (Figs. **1G**, **H**) (Table **S6**). Interestingly, a significant increase in the Aβ42/Aβ40 ratio was demonstrated after prenatal stress in 12-month-old (Hp: *p* < 0.0001; Cx: *p* < 0.0001), 15-month-old (Hp: *p* < 0.0001; Cx: *p* < 0.0001), and 18-month-old (Hp: *p* < 0.0001; Cx: *p* < 0.0001) KI mice compared to age-matched WT PS mice (Figs. **1G**, **H**) (Table **S6**). Importantly, compared with the control treatment, PS treatment increased the Aβ42/Aβ40 ratio in 15-month-old (Cx: *p* = 0.009630) and 18-month-old (Hp: *p* = 0.00048; Cx: *p* = 0.007614) KI mice (Figs. **1G**, **H**).

### 3.4 Age-dependent Impact of PS on the mRNA Expression of Pro- and Anti-inflammatory Factors in the Hippocampi and Frontal Cortices of Normal Aging (WT) and Knock-in APP^NL-F/NL-F^ Mice

**Fig. (2):**
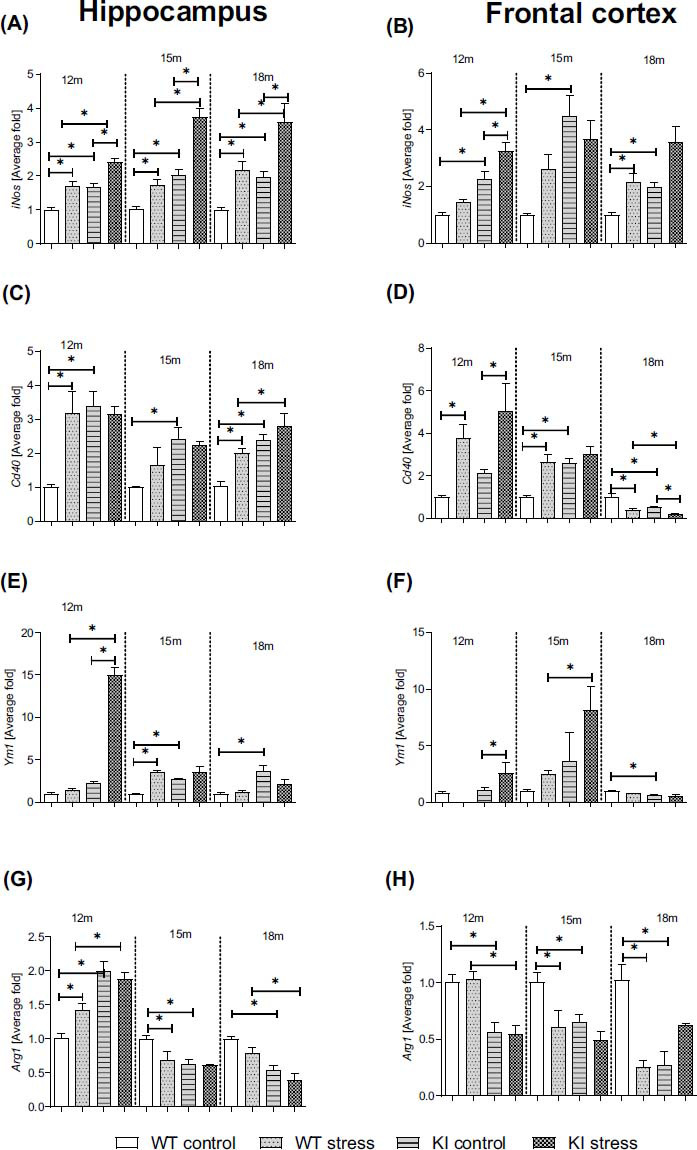
Age-dependent impact of the prenatal stress on the mRNA expression of inducible nitric oxide synthase (*iNOS*; average fold, **A**, **B**), cluster of differentiation 40 (*Cd40*; average fold; **C**, **D**), chitinase-like protein 3 (*Ym1*; average fold, **E**, **F**) and arginase-1 (*Arg1*; average fold, **G**, **H**) in the hippocampi and frontal cortices of 12-, 15-, and 18-month-old wild-type (WT) and APP^NL-F/NL-F^ (KI) mice. ANOVA with Duncan’s *post hoc* test (*p < 0.05, n = 3-4 mice in each group, error bars are standard deviations).

Importantly, qRT-PCR analysis of KI mice revealed upregulated *iNos* gene expression at the ages of 12 (Hp: *p* = 0.000623; Cx: *p* = 0.001689), 15 (Hp: *p* = 0.002290; Cx: *p* = 0.002001), and 18 months (Hp: *p* = 0.029315; Cx: *p* = 0.003155) compared to that in age-matched WT controls (Figs. **2A**, **B**) (Table **S3**). An effect of PS was observed for both genotypes. PS led to increased *iNos* expression in WT (Hp: 12: *p* = 0.000532; 15: *p* = 0.017546; 18: *p* = 0.013979; and Cx: 18: *p* = 0.029346) and KI mice (Hp: 12: *p* = 0.000516; 15: *p* = 0.000203; 18: *p* = 0.001683; and Cx: 12: *p* = 0.005634) compared to age-matched controls. Importantly, the effect of PS on KI mice was greater than that on WT mice (Hp: 12: *p* = 0.000606; 15: *p* = 0.000095; 18: *p* = 0.003365; Cx: 12: *p* = 0.000141) (Figs. **2A**, **B**) (Table **S3**).

Regarding the differences between the control KI and WT mice, significant increases in *Cd40* gene expression were observed at 12 (Hp: *p* = 0.000873), 15 (Hp: *p* = 0.005062; Cx: *p* = 0.009078), and 18 months (Hp: *p* = 0.001230) (Figs. **2C**, **D**) (Table **S3**). Additionally, the *Cd40* expression level was increased after the prenatal stress procedure in the hippocampi (12: *p* = 0.001528; 18: *p* = 0.009258) and frontal cortices (12: *p* = 0.033670; 15: 0.010567) of in WT mice and in the frontal cortices of KI animals (Cx: 12: *p* = 0.025642). In turn, in the frontal cortex samples from 18-month-old KI mice, the expression of *Cd40* was decreased (*p* = 0.000344), and PS also led to a decrease in Cd40 expression (WT: *p* = 0.000137; KI: *p* = 0.006296) (Figs. **2C**, **D**) (Table **S3**).

As illustrated in Figs. (**2E** and **2F**), *Ym1* expression in the hippocampus was upregulated (15-month-old: *p* = 0.008304; 18-month-old: *p* = 0.025063), and Ym1 expression in the frontal cortex was downregulated (18-month-old: *p* = 0.019597) in KI mice compared to age-matched WT mice. The stress procedure during the prenatal period significantly increased *Ym1* expression in the hippocampi of 15-month-old WT offspring (*p* = 0.001010) (Figs. **2E**, **F**). Prenatal stress also upregulated *Ym1* expression in the hippocampi of 12-month-old offspring (*vs.* WT PS: *p* = 0.000092; *vs.* KI: *p* = 0.000210) and in the frontal cortices of 12-month-old (*vs.* KI: *p* = 0.012209) and 15-month-old (*vs.* WT: *p* = 0.029870) offspring (Figs. **2E**, **F**) (Table **S3**).

In the subsequent series of experiments, notable reductions were observed in the expression levels of the *Arg1* gene within the hippocampus (15-month-old: *p* = 0.003461; 18-month-old: *p* = 0.001577) and frontal cortex (12-month-old: *p* = 0.02057; 15-month-old: *p* = 0.027409; 18-month-old: *p* = 0.004546) (Figs. **2G**, **H**) (Table **S3**) compared to those in age-matched WT offspring.

Prenatal stress affected the hippocampi of 15-month-old (*p* = 0.008635) and the frontal cortices of 15-month-old (*p* = 0.020190) and 18-month-old (*p* = 0.004348) offspring (Figs. **2G**, **H**). Moreover, after prenatal stress, KI mice expressed *Arg-1* at lower levels than WT PS mice (Hp: 18 months old: *p* = 0.004782; Cx: 12 months old: *p* = 0.001611) (Table **S3**).

### 3.5 Age-dependent Impact of PS on **the** Pro- and Anti-inflammatory Cytokines Protein Levels in the Hippocampi and Frontal Cortices in Normal Aging (WT) and Knock-in APP^NL-F/NL-F^ Mice

Based on the well-known finding that cytokines are key components of neuroinflammation, in our study, we decided to measure the protein levels of several crucial pro- (IL-1β) and anti-inflammatory cytokines (IL-10 and TGF-β) involved in the inflammatory response in brain homogenates from the hippocampi and frontal cortices of normal aging (WT) and KI mice.

**Fig. (3):**
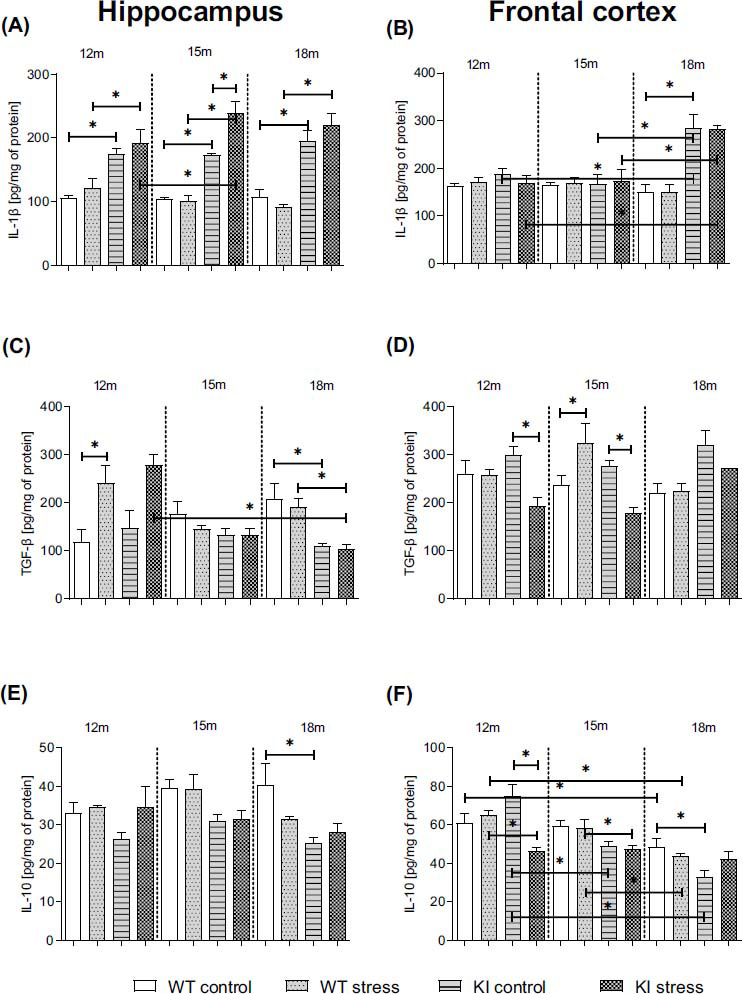
Age-dependent impact of prenatal stress on the level of interleukin 1β (Il-1β; pg/mg protein; **A**, **B**) transforming growth factor β (TGF-β; pg/mg protein; **C**, **D**) and on interleukin 10 (IL-10; pg/mg protein; **E**, **F**) in the hippocampi and frontal cortices of 12-, 15-, and 18-month-old wild-type (WT) and APP^NL-F/NL-F^ (KI) mice. ANOVA with Duncan’s *post hoc* test (*p < 0.05, n = 3-4 mice in each group, error bars are standard deviations).

As shown in Figs. **3A** and **3B**, 12- and 15-month-old KI animals exhibited elevated levels of IL-β in the hippocampus (*p* = 0.001079; *p* = 0.001188, respectively). In 18-month-old KI mice, this effect was observed in both analyzed brain areas (Hp: *p* = 0.000095; Cx: *p* = 0.000016). Moreover, after PS, compared with WT mice, KI animals were characterized by an increased level of IL-β in the hippocampus at any age (12-: *p* = 0.000814; 15: *p* < 0.0001; 18: *p* < 0.0001). Statistical analysis revealed that prenatal stress led to a significantly greater increase in IL-β production in the hippocampus of 15-month-old transgenic mice than in that of KI controls (*p* = 0.002141) and 12-month-old KI PS mice (*p* = 0.022466). An age-dependent impact of stress was found in the frontal cortex of both control and PS KI mice. The level of IL-β in 18-month-old KI mice was higher than that in 12-month-old (control: *p* = 0.000273; PS: *p* < 0.00001) and 15-month-old (control: *p* = 0.000070; PS: *p* = 0.000100) mice (Table **S6**).

Next, ELISA revealed that, compared to that in age-matched WT animals, the TGF-β concentration in the hippocampus was lower in 18-month-old KI control offspring (Hp: *p* = 0.007276) (Fig. **3C**). Prenatal stress resulted in increased TGF-β levels in the hippocampi of 12-month-old WT mice (*p* = 0.001202) and the frontal cortices of 15-month-old WT mice (*p* = 0.015256) (Fig. **3D**). In turn, compared with age-matched KI mice, KI PS mice were characterized by lower levels of the analyzed cytokines in the frontal cortex (12-: *p* = 0.003277; 15: *p* = 0.007264). Moreover, in the hippocampi of 18-month-old KI PS animals, the level of TGF-β was lower than that in the hippocampi of 12-month-old KI PS (*p* = 0.00000023) and 18-month-old WT PS (*p* = 0.018284) animals (Figs. **3C**, **3D**) (Table **S6**).

In the next set of experiments, we reported a significant decrease in IL-10 levels in 18-month-old KI animals (Hp: *p* = 0.004281; Cx: *p* = 0.008506) compared to age-matched WT offspring (Figs. **3E**, **F**). PS affected only the frontal cortex of 12-month-old (*p* = 0.000026) KI mice (Fig. **3F**). In the frontal cortex of adult WT and KI control female offspring, we found that the IL-10 level was higher in 12-month-old mice than in 15- (KI: *p* = 0.000054) and 18-month-old (WT: *p* = 0.033088; KI: *p* = 0.007023) genotype-matched mice (Figs. **3E**, **F**) (Table **S6**). Compared with WT PS, stress during pregnancy led to a decreased IL-10 level in KI mice at the same age (12: *p* = 0.002514; 15: *p* = 0.045440). Moreover, the level of IL-10 in 18-month-old WT PS mice was lower than that in 15- (*p* = 0.013110) and 12-month-old (*p* = 0.000609) mice. Our study demonstrated age-dependent changes in pro- and anti-inflammatory cytokine release in various brain areas, which may be responsible for the dysregulation of cytokine balance and prolongation of the inflammatory response, which was more apparent in KI mice.

### 3.6 Age-dependent Impact of PS on the NF-κB and A20 Protein Levels in the Hippocampi and Frontal Cortices of Normal Aging (WT) and Knock-in APP^NL-F/NL-F^ Mice

NF-κB is a central mediator of NLRP3 inflammasome activation and acts by inducing the synthesis and release of proinflammatory cytokines (IL-1β and IL-18). Currently, A20 is considered a negative regulator of the NF-κB-dependent inflammatory response. Thus, we examined both the protein levels of A20 and the phosphorylation level of the p65 NF-κB subunit in brain homogenates from the hippocampi and frontal cortices of normal aging (WT) and KI mice.

**Fig. (4):**
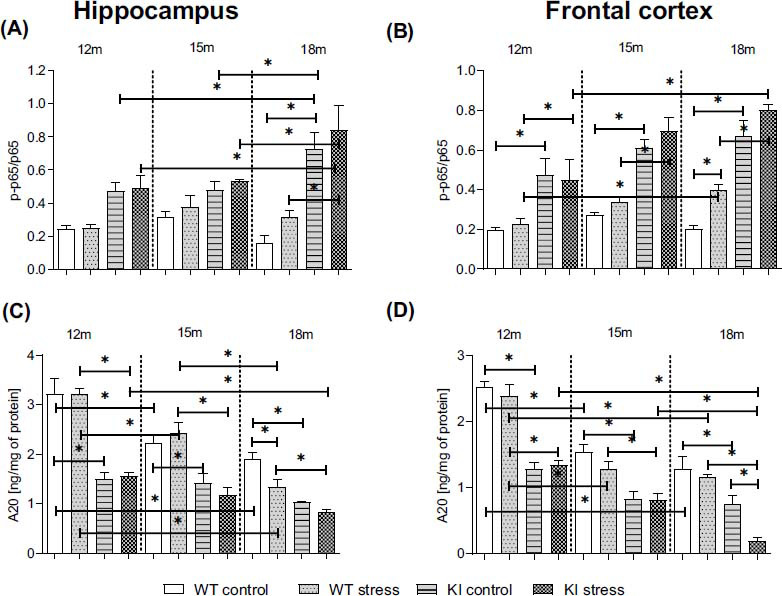
Age-dependent impact of prenatal stress on the phospho-p65/total p65 ratio (**A**, **B**) and the phosphorylation of TNF-α-induced protein 3 (A20; ng/mg of protein; **C**, **D**) in the hippocampi and frontal cortices of 12-, 15-, and 18-month-old wild-type (WT) and APP^NL-F/NL-F^ (KI) mice. ANOVA with Duncan’s *post hoc* test (*p < 0.05, n = 3-4 mice in each group, error bars are standard deviations).

Examination of the brain areas in adult KI mice by ELISA revealed a significant increase in the phospho-p65/total p65 ratio at any age in control (12 (Cx): *p* = 0.001769), 15 (Cx: *p* = 0.000199), and 18-month-old (Hp: *p* = 0.000036; Cx: *p* = 0.000019)) mice compared to that in age-matched WT offspring (Figs. **4A**, **B**) (Table **S4**). Moreover, there was an increase in the phospho-p65/total p65 ratio in prenatally stressed KI mice (12 (Cx: *p* = 0.016424), 15 (Cx: *p* = 0.000097), and 18-month-old (Hp: *p* = 0.000062; Cx: *p* = 0.000033)) mice compared to that in age-matched WT animals treated in the same way. The phospho-p65/total p65 ratio was higher in 18-month-old KI animals than in 12-month-old (*p* = 0.02889) and 15-month-old genotype-matched mice (*p* = 0.029767). In the case of the WT offspring, stress during pregnancy had an effect only on the frontal cortex at 18 months of age (*p* = 0.022852). Moreover, we found an age-dependent increase in phospho-p65/total p65 in 18-month-old KI PS mice compared to 12-month-old (Hp: *p* = 0.002630; Cx: *p* = 0.000098) and 15-month-old (Hp: *p* = 0.029767) offspring and in 18-month-old WT PS animals (Cx: *vs.* 12-month-old: *p* = 0.041897) (Figs. **4A**, **B**) (Table **S4**).

Next, we demonstrated significant differences in A20 protein levels between WT and KI mice; a significant decrease was observed in both the analyzed brain areas of control mice at the ages of 12 (Hp: *p* < 0.00001; Cx: *p* = 0.000022), 15 (Hp: *p* = 0.002080; Cx: *p* = 0.00351), and 18 months (Hp: *p* = 0.001230; Cx: *p* = 0.006543) (Figs. **4C**, **D**); and in the prenatally stressed KI mice aged 12 (Hp: *p* = 0.000018; Cx: *p* = 0.00064), 15 (Hp: *p* < 0.00001; Cx: *p* = 0.014122), and 18 months (Hp: *p* = 0.039851; Cx: *p* = 0.002013) compared to age-matched WT PS mice (Figs. **4C**, **D**). Moreover, strong effects of prenatal stress were observed in the frontal cortices of KI offspring at 18 months of age (*p* = 0.000924) (Fig. **4D**) and in the hippocampi of WT animals (*p* = 0.031372) (Fig. **4C**) (Table **S4**).

Interestingly, we observed lower A20 levels in both analyzed brain areas of 15-month-old (Hp: *p* = 0.000248; Cx: *p* = 0.000038) and 18-month-old (Hp: *p* < 0.0001; Cx: *p* = 0.000035) WT mice than in 12-month-old WT mice (Figs. **4C**, **D**) (Table **S4**). A similar effect was found after the prenatal stress procedure in WT offspring at the ages of 15 (*p* = 0.001599; *p* = 0.000053) and 18 months (*p* < 0.0001; *p* = 0.000022). In addition, our results showed that 18-month-old KI PS mice were characterized by a lower level of A20 than 12-month-old (Hp: *p* = 0.006728; Cx: *p* = 0.000018) and 15-month-old (Cx: *p* = 0.001089) mice (Figs. **4C**, **D**). The same effect was observed in the hippocampi of the oldest WT PS mice and 15-month-old age-matched mice (*p* = 0.000113) (Fig. **4C**). Thus, not only age but also the KI procedure significantly modulates the NF-κB-A20 pathway in the brain.

### 3.7 Age-dependent Impact of PS on the NLRP3 Inflammasome Components Protein Levels in the Hippocampi and Frontal Cortices of Normal Aging (WT) and Knock-in APP^NL-F/NL-F^ Mice

The NLRP3 complex is composed of the intracellular receptor NLR, the protein ASC, and the inactive procaspase-1. When the NLRP3 inflammasome is activated, the inactive forms of proinflammatory cytokines interleukin-1β and interleukin-18 are transformed into active cytokines. We estimated the protein levels of the NLRP3 inflammasome components (NLRP3, caspase-1, and ASC) in brain homogenates from the hippocampi and frontal cortices of normal aging (WT) and KI mice.

**Fig. (5):**
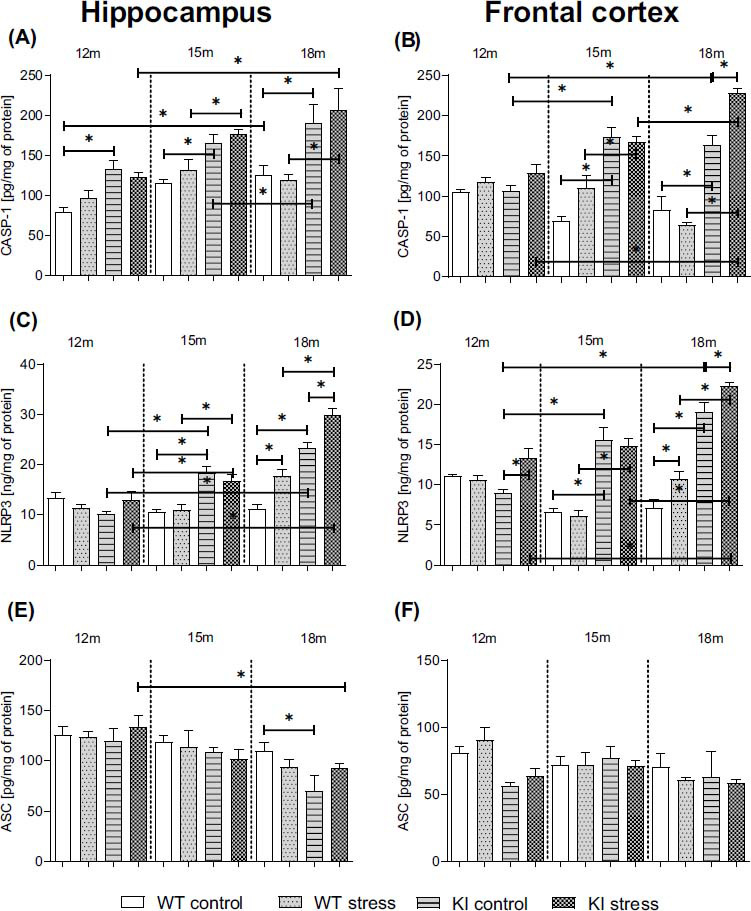
Age-dependent impact of prenatal stress on components of the NLRP3 inflammasome: Caspase1 (CASP-1; pg/mg of protein; **A**, **B**) nucleotide-binding domain, leucine-rich-containing family, pyrin domain-containing-3 (NLRP3; ng/mg of protein; **C**, **D**), and apoptosis-associated speck-like protein containing a CARD (ASC; pg/mg of protein; **E**, **F**) protein levels in the hippocampi and frontal cortices of 12-, 15-, and 18-month-old wild-type (WT) and APP^NL-F/NL-F^ (KI) mice. ANOVA with Duncan’s *post hoc* test (*p < 0.05, n = 3-4 mice in each group, error bars are standard deviations).

We observed that the Casp-1 level was elevated in both brain areas of the KI mice at the ages of 12 (Hp: *p* = 0.022949), 15 (Hp: *p* = 0.034952; Cx: *p* = 0.00017), and 18 months (Hp: *p* = 0.005799; Cx: *p* = 0.00018) (Figs. **5A**, **B**) (Table **S4**). Moreover, the Casp-1 level observed in the 18-month-old mice was significantly higher than that observed in the 12-month-old (WT: Hp: *p* = 0.046045; KI: Cx: *p* = 0.000435) and 15-month-old (KI: Hp: *p* = 0.011682) genotype-matched mice (Figs. **5A**, **B**). Similarly, compared with those of 12-month-old mice, the frontal cortices of 15-month-old control mice were characterized by elevated Casp-1 levels (*p* = 0.000085) (Fig. **5B**). The effects of prenatal stress were specifically noted only in KI mice, manifesting as a further elevation in Casp-1 levels in the frontal cortices of 18-month-old (*p* = 0.000104) mice. Importantly, the Casp-1 level in the 18-month-old KI PS mice was significantly higher than that in the 12-month-old (Hp: *p* = 0.000588; Cx: *p* = 0.000031) and 15-month-old (Cx: *p* = 0.000151) KI PS animals. Moreover, after PS, the Casp-1 levels were further intensified 15-month-old (Hp: *p* = 0.0485367; Cx: *p* = 0.000489) and 18-month-old (Hp: *p* = 0.000414; Cx: *p* = 0.00012) KI mice, compared with WT mice of the same age.

As shown in Figs. **5C** and **5D**, we observed an increase in the hippocampal and cortical NLRP3 protein levels in control KI mice at the ages of 15 (Hp: *p* = 0.000201; Cx: *p* = 0.000017) and 18 months (Hp: *p* = 0.000020; Cx: *p* = 0.000017) in comparison to age-matched WT mice (Figs. **5C**, **D**) (Table **S4**). Moreover, in KI mice, we observed an age-dependent increase in NLRP3 levels in 15-month-old (Hp: *p* = 0.000123; Cx: *p* = 0.000046) and 18-month-old (Hp: *p* = 0.000018; Cx: *p* = 0.000019) mice compared to 12-month-old genotype-matched mice. The stress procedure during the prenatal period significantly increased NLRP3 levels in the WT (18-month-old: Hp: *p* = 0.001158; Cx: *p* = 0.011228) and KI offspring (12-month-old: Cx: *p* = 0.003251; 18-month-old: Hp: *p* = 0.000685; Cx: *p* = 0.012492) compared to genotype-matched and age-matched mice (Figs. **5C**, **D**). Interestingly, the effect of PS on KI mice was greater than that on WT animals at the ages of 15 (*p* = 0.002893; *p* = 0.000018) and 18 months (*p* = 0.000055; *p* = 0.000022). We also observed an age-dependent increase in NLRP3 levels in the hippocampi and frontal cortices of 18-month-old KI mice subjected to prenatal stress (*vs*. 12-month-old Hp: *p* = 0.000023; Cx: *p* = 0.000031; *vs*. 15-month-old Hp: *p* = 0.000033; Cx: *p* = 0.000054) (Figs. **5C**, **D**) (Table **S4**).

We detected a decrease in the level of the ASC protein in the hippocampi of 18-month-old (*p* = 0.017291) KI mice (Fig. **5E**). Additionally, compared with 12-month-old genotype-matched animals, PS-treated mice presented a decrease in ASC levels (*p* = 0.022437) (Figs. **5E** and **5F**). Thus, the knock-in procedure leads to age-dependent increases in CASP-1 and NLRP3 protein levels, while PS potentiated the observed changes mainly in the oldest group of KI mice.

### 3.8 Age-dependent Impact of PS on the Senescence-associated Proteins p53, p16, and pRb in the Hippocampi and Frontal Cortices of Normal Aging (WT) and Knock-in APP^NL-F/NL-F^ Mice

Rigorous comparisons of aging- and senescence-associated cellular changes in the murine brain are rare. Although cellular senescence has been demonstrated to play an important role in age-related diseases (*e.g*., AD), the causes of this process under either physiological or pathological conditions have not been determined [42]. Recently, data have suggested that a prolonged proinflammatory response might lead to the dysfunction of various proteins, including senescence molecules, in the brain. Therefore, in the last part of our study, we decided to measure the levels of the p53, p16, and pRb molecules in brain homogenates from the hippocampi and frontal cortices of normal aging (WT) and KI mice.

**Fig. (6):**
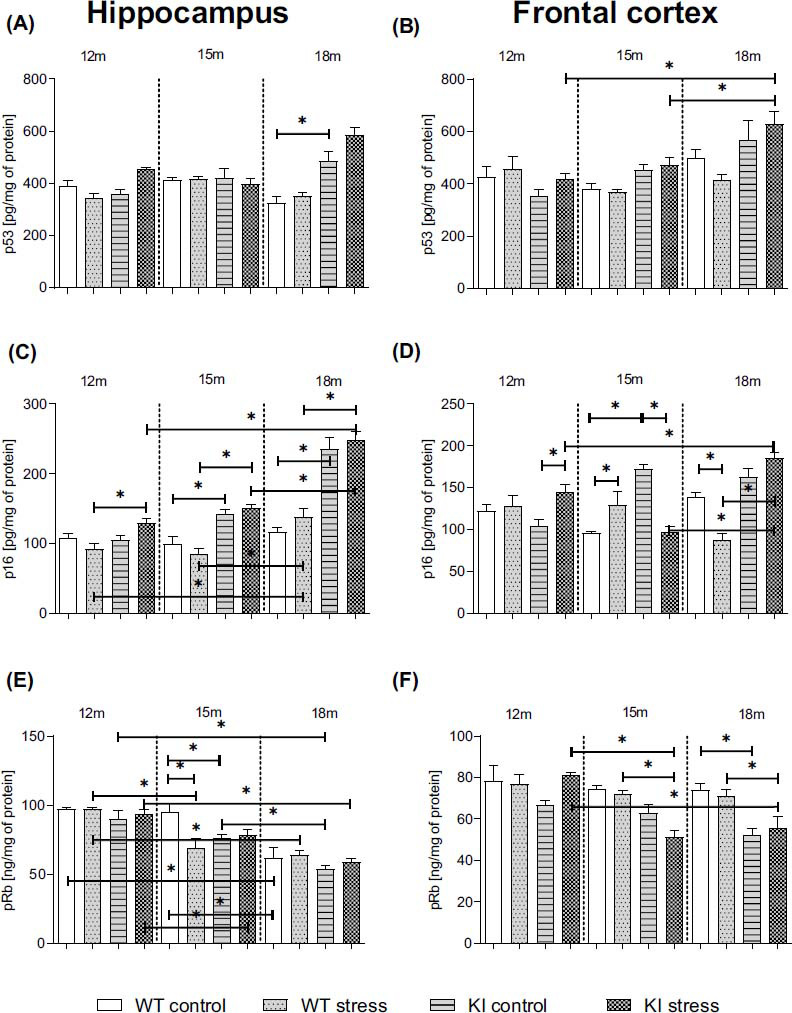
Age-dependent impact of prenatal stress on the levels of the senescence-associated proteins p53 (pg/mg of protein; **A**, **B**), p16 (pg/mg of protein; **C**, **D**), and pRb (ng/mg of protein; **E**, **F**) in the hippocampi and frontal cortices of 12-, 15-, and 18-month-old wild-type (WT) and APP^NL-F/NL-F^ (KI) mice. ANOVA with Duncan’s *post hoc* test (*p < 0.05, n = 3-4 mice in each group, error bars are standard deviations).

Examination of the samples derived from the adult mice by ELISA revealed a significant increase in the p53 protein level in the hippocampus of 18-month-old KI animals compared to WT animals (*p* = 0.000141; Fig. **6A**) (Table **S5**). An age-dependent stimulatory effect of prenatal stress on p53 levels was observed in the frontal cortices of 18-month-old KI mice compared to 15-month-old (*p* = 0.005193) and 12-month-old (*p* = 0.000491) mice (Fig. **6B**) (Table **S5**).

As shown in Figs. **6C** and **6D**, we observed increased p16 levels in both the hippocampi (15-month-old: *p* = 0.004470; 18-month-old: *p* = 0.000027) and frontal cortices (15-month-old: *p* = 0.000020; 18-month-old: *p* = 0.000012) of KI mice compared to age-matched WT mice. The promotive effect of prenatal stress on p16 levels was shown in the frontal cortices of 12-month-old (*p* = 0.004980) KI offspring, KI control, and 15-month-old WT PS (*p* = 0.020298) adult offspring compared with age-matched controls (Figs. **6C**, **D**) (Table **S5**). Stress during pregnancy led to an increase in the level of p16 in the hippocampi and frontal cortices of KI mice compared with WT PS mice at the same age (Hp: 12-month-old: *p* = 0.013034; 15-month-old: *p* < 0.00001; 18-month-old: *p* < 0.00001; Cx: 18-month-old: *p* = 0.000012) (Figs. **6C**, **D**). Moreover, both analyzed brain areas of 18-month-old PS KI mice were characterized by a higher level of p16 than that in 12-month-old (*p* = 0.000062; Cx: *p* = 0.003895) and 15-month-old (Hp: *p* = 0.000027; Cx: *p* = 0.000018) KI controls (Figs. **6C**, **D**). Similarly, in the case of the WT mice, we observed similar results in the hippocampi of 18-month-old mice and 15- (*p* = 0.000666) and 12-month-old (*p* = 0.002494) offspring (Figs. **6C**, **D**) (Table **S5**).

Finally, we measured the age-dependent changes in the pRb protein levels. The knock-in procedure decreased the level of pRb in the hippocampi of 15-month-old mice (*p* = 0.006505) and in the frontal cortices of 18-month-old (*p* = 0.000753) *vs.* age-matched WT controls (Figs. **6E**, **F**) (Table **S5**). Additionally, in the hippocampi of 18-month-old mice, we observed an age-dependent decrease in pRb levels compared to those in the hippocampi of 15-month-old (KI: *p* = 0.001116; WT: *p* = 0.000027) and 12-month-old (KI: *p* < 0.0001; WT: *p* = 0.000020) animals. Stress during pregnancy led to a decreased pRb level in the hippocampi of 15-month-old WT mice (*p* = 0.000269). Moreover, after the PS procedure, we observed an age-dependent decrease in pRb levels in 15-month-old (Hp: KI: *p* = 0.000742; WT: *p* = 0.000123; Cx: KI: *p* = 0.000491) and 18-month-old (Hp: KI: *p* = 0.000028; WT: *p* = 0.000028; Cx: KI: *p* = 0.005193) mice compared to 12-month-old genotype-matched mice (Figs. **6E**, **F**) (Table **S5**). The effect of PS on the frontal cortex was greater in (15- (*p* = 0.001406) and 18-month-old (*p* = 0.012062) KI mice than in the WT mice. Overall, we demonstrated an increase in p16 and a decrease in pRb protein levels in aging KI mice compared with age-matched controls and the differential impact of stress on these parameters (Figs. **6E**, **F**) (Table **S5**).

## 4 DISCUSSION

Brain aging is a complex process involving genetic, neurodevelopmental, and environmental factors. Inherent features of this process include prolonged inflammation, deficits in the resolution of inflammation (RoI), cellular senescence, and the development of a senescence-associated secretory phenotype (SASP). Persistent unresolved inflammation and inflammation associated with age (“inflame aging”) are thought to exacerbate the progression of cognitive and behavioral decline observed in both physiological aging and age-dependent AD pathophysiology [43, 44]. In our study, we investigated whether differences in the course of inflammation resolution (RoI) correlated with cognitive changes in a genetic animal model of AD (KI) and physiological aging in WT mice. Moreover, we compared the impact of prenatal stress (PS), a potent neurodevelopmental immune challenge, on the course of RoI in the abovementioned animal models.

We found that, in the Morris water maze test, spatial learning ability was markedly disrupted in 18-month-old prenatally stressed KI mice, which indicates that the brain areas affected by aging can still be conditioned by factors operating during neurodevelopment [45]. Prenatal stress may influence spatial memory *via* multiple molecular mechanisms. Additionally, the knock-in procedure itself may also be a meaningful factor in determining cognitive deficits. Indeed, in the probe trial, we observed diminished distance traveled in the NE zone by KI mice at the age of 15 months, while in the older 18-month-old animals, spatial memory deficits were also revealed, as the latency to the first entry to the platform zone was increased compared to the that of age-matched WT animals. It should be noted that these deficits could be observed in the oldest WT and prenatally stressed offspring. These results strongly support previous observations in which deficits in escape latency and path efficiency to the first entry to the platform zone, as well as the number of line crossings in this area, were demonstrated in aging KI mice [38]. We also performed the open-field test, in which several behavioral parameters provide a qualitative and quantitative measurement of movement activity in mice [46]. Although aging negatively affected these measured parameters, prenatal stress caused only a transient reduction in distance movement, mobility, and velocity in 12-month-old KI mice compared to age-matched WT mice. Notably, motor dysfunction is observed in Alzheimer's disease patients, including mildly affected individuals [47]; however, it is still not known whether this dysfunction is due to diminished motivation for physical activity or simply reflects the inability to move quickly [present study, 38]. In addition, differences in distance traveled were present between 15-month-old and older animals with obvious AD pathology. Additionally, other authors reported a reduction in open-field activity in APP^NL-G-F^ mice [45]. Thus, our behavioral study provided evidence that aging is an important factor responsible for deficits in spatial learning and memory, as well as behavioral activity measured in the open field test in KI mice. Prenatal stress aggravated cognitive deficits in the oldest group of KI animals; however, in the open field test, adverse effects of stress could be detected only in aging offspring. Our study also showed that some behavioral parameters are more prone to stress-induced changes than others.

The aging brain shows a progressive decrease in weight and volume, secondary to reduced neuronal arborization and loss of myelin [48], and glial cells increase and mediate various brain immune responses. In the brain, the resolution of inflammation (RoI) is a highly regulated process mediated by the pro-resolving activation of the FPR2 receptor [49]. However, whether age-dependent disturbances in the RoI can trigger cognitive deficits in KI mice and whether prenatal stress may have an impact on these changes remain unclear. To address this issue, we conducted biochemical and molecular studies in KI offspring at 12, 15, and 18 months, both before and after behavioral deficits appeared, and compared the results with those in WT animals of the same age.

The main finding in the present work was the age-dependent increase in FPR2 levels in the hippocampi and frontal cortices of KI mice. Moreover, we found that prenatal stress further elevated the level of this receptor in the frontal cortices of 18-month-old KI mice. We unexpectedly observed no changes in FPR2 levels during physiological aging, while the impact of stress was present only in the hippocampi of 18-month-old WT mice. FPR2 is considered the most promiscuous member of the FPR family because it chemically recognizes various endogenous and exogenous ligands [50-52]. A large body of evidence suggests that FPR2 mediates Aβ_1-42_-elicited proinflammatory responses. In the present study, we reported a dramatic increase in the human amyloid β_1-42/1-40_ ratio in both brain areas of KI mice. Moreover, maternal stress further enhanced this ratio in the oldest groups of offspring. Amyloid precursor protein (APP) in the brain promotes amyloidogenic proteolytic cleavage and the amyloid formation pathway, resulting in Aβ_1-38_, _1-40,_ and _1-42_, with the latter showing a greater tendency to aggregate. This early-stage pathogenic process induces a proinflammatory cascade that over-activates astrocytes and microglia [44, 53]. As observed in the present study, this overactivation precedes the appearance of cognitive and behavioral deficits. FPR2 also mediates anti-inflammatory effects when it interacts with ligands with pro-resolving properties. Among them, lipoxin A4 (LXA4) is the most specific [51]. Its expression has been detected in neural stem cells, neurons, astrocytes, and microglia [54, 55]. LXA4 exerts a protective effect through its beneficial impact on neuronal survival and enhancement of microglial phagocytic and anti-inflammatory potential [56]. Interestingly, LXA4 was found to reduce Aβ levels and improve cognition in mouse models of Alzheimer’s disease [57]. Mechanistically, the relationship between the resolution pathway and cognition is based mainly on the correlation of LXA4 levels with the aging brain [58]. In the present study, we showed that, in the frontal cortex, the level of LXA4 decreases with age in WT mice; however, in KI offspring, the deficits were much more pronounced and present in both brain areas. Interestingly, we also detected a significant reduction in Annexin 1 (ANXA1) levels in 12-month-old male KI mice. Notably, ANXA1 binding with FPR2 is implicated in mediating phagocytosis and neuroprotective effects in the brain during aging [59, 60]. However, ANXA1 also mediates glucocorticoid anti-inflammatory action that can suppress microglial activation and reduce blood-brain barrier leakage [61]. It has been found that treatment with recombinant human ANXA1 (hrANXA1) reduced amyloid-β. Moreover, the beneficial effects of hrANXA1 *in vivo* by restoring efficient blood-brain barrier function and decreasing amyloid-β and tau pathology in 5xFAD mice and Tau-P301L mice have been demonstrated. The systemic anti-inflammatory properties of hrANXA1 were also observed in 5xFAD mice, increasing IL-10 and reducing TNF-α expression. Hence, the diminished ANAX1 level in our study in KI mice may limit its anti-inflammatory properties *via* FPR2 activation. Thus, the increased level of FPR2 in KI mice may result from the proinflammatory environment, which drives the expression of FPR2 [62]. Hence, we consider the strong age-dependent decrease in LXA4 levels in KI mice and the disturbances in homeostatic FPR2 activation (by pro-inflammatory and pro-resolving ligands) to be crucial factors leading to changes in the profile of FPR2 activation, likely contributing to the development of RoI deficits.

At the molecular level, FPR2 activation also determines the brain cell type involved. In our previous study, we showed the crucial role of microglial FPR2 expression in the modulation of the RoI [41, 49]. Hence, in the present research, we assessed the potential impact of RoI dysfunction in KI mice on microglia over time. We noticed a significant increase in *iNOS* expression in both examined brain structures in KI mice compared to WT mice and this change, mainly in the hippocampus, was potentiated by prenatal stress. Stress also increased *iNos* mRNA expression in WT mice. Moreover, a reduction in *Arg* expression in both brain structures was found in KI mice. Maternal stress also modulated *Ym1* and *Cd40* gene expression in KI mice compared to WT mice, indicating that changes in the neurodevelopmental environment affect the microglial activation profile [63] and translate to an entirely different trajectory of microglia in adulthood in KI and WT mice. In our study, we also detected a long-lasting increase in IL-1β levels in the hippocampi of 12-month-old KI mice and in the frontal cortices of 15-month-old mice. Simultaneously, in the hippocampus, the potentiating effect of stress during pregnancy on the level of this cytokine and, thus, on proinflammatory brain status in KI offspring was evident. Recent work has shown that in the progression of neurodegeneration, central events involve the NF-κB and NLRP3 pathways and the activation of microglia by proinflammatory cytokines and Aβ aggregation [43]. The NLRP3 inflammasome is a complex composed of several proteins, including the NLRP3 subunit, procaspase-1, and the apoptosis-associated speck-like protein containing a caspase recruitment domain (ASC). Activation of the NLRP3 inflammasome occurs in two stages, involving the assembly of inactive NLRP3, ASC, and procaspase-1 into a complex. This complex then catalyzes the conversion of procaspase-1 into caspase-1, leading to the production and release of mature proinflammatory cytokines, particularly IL-1β [64].

A striking observation from the present study was the enormous age-dependent increase in caspase-1 levels in the KI hippocampus and frontal cortex compared to those in age-matched WT mice. Additionally, we observed an increase in the levels of the NLRP3 components in KI offspring. In the oldest offspring, stress intensified these changes, indicating its prolonged impact on NLRP3 activation. Moreover, for the first time, we demonstrated an increase in the phosphorylation of the p65 subunit of the NF-κB transcription factor in both structures in 12-month-old KI mice, which was accompanied by a progressive, age-dependent decrease in the level of A20 in KI offspring compared to that in age-matched WT mice. Similarly, changes also occur during physiological aging but are less pronounced. Thus far, A20 has been reported to inhibit inflammation by regulating the NF-κB pathway and counteracting the cytotoxic effects of proinflammatory cytokines [65, 66]. However, the physiological importance of A20 is not limited to its role in NF-κB activation and IL-1β release [67]. Interestingly, A20 may also exert a self-protective effect on the senescence of brain cells induced by the high release of various proinflammatory factors [68]. Therefore, based on these findings, we believe that the disruption of NF-κB-NLPR3 signaling mediated by the lack of A20 might be one of the molecular mechanisms responsible for exacerbating RoI dysfunction and probably cellular senescence in the brain [69]. This process classically involves two main regulatory pathways: the p53-pRB pathway and the p16-pRB pathway. In the present study, the p53 pathway was activated in the hippocampi of 18-month-old KI mice, while prenatal stress intensified this activation in the frontal cortex. Frequently, the p53 pathway is activated by DNA damage, which can include telomere damage [69]. However, the specificity of this process is strongly dependent on the genetic background and/or its modifications, as well as on the proliferation ability of cells, which could also be affected by age [70]. Moreover, some data suggest that telomere shortening does not influence aging or lifespan in mice [71]. In addition to the increased p53 pathway activation, KI mice exhibited a substantial increase in the p16 protein level and a decrease in the pRb protein level compared to those in WT mice in both brain areas. The most significant age-dependent changes occurred in animals aged 15 months or older. Thus far, Baker *et al*. [72] have indicated that removing p16-positive cells benefits the surrounding tissue, delays aging, and increases the lifespan of mice. Indeed, the downregulation of p16 expression, primarily in microglia, diminishes the expression of SASP factors and improves cognitive function in aged mice [73]. In contrast, amyloid β accumulation in the brain increases the expression of senescence markers in glial cells. In addition to senescence, the p16 protein may be crucial for shifting microglial/peripheral macrophage polarization to inflammatory phenotypes [74]. Nevertheless, a previous study revealed that an FPR2 antagonist (WRW4) inhibits senescence induced by amyloid β_1-42_ [75]. Considering the data presented above, the involvement of FPR2 in RoI processes, cellular aging, and potentially in the modulation of cognitive deficits has been proposed, although the underlying mechanisms remain unclear.

Previous studies have demonstrated that TGF-β signaling is involved in aging processes and cellular senescence [76] and impacts brain growth and function, neuronal patterning, microglial development, and neurogenesis [77]. In the present study, we showed dynamic changes in TGF-β levels in the brain areas of the oldest KI offspring. Additionally, prenatal stress affected TGF-β levels in 12- and 15-month-old KI mice. It is difficult to interpret these findings because TGF-β signaling elicits complex effects (beneficial or detrimental) in the brain [76]. While elevated TGF-β levels in brain tissue and cerebrospinal fluid (CSF) have been observed in Alzheimer's disease patients [78, 79], the impairment of TGF-β signaling in neurons is postulated to be a standard feature of AD [80]. Indeed, TGF-β signaling dysregulation in neurons potentiates amyloid β accumulation [80] and leads to alterations in microglial function, triggering pathogenic changes [81-83]. Therefore, a decrease in the level of TGF-β in 18-month-old KI mice may be responsible for the loss of brain cell function in an aging brain. However, the role of TGF-β in the brain is generally cell type dependent, and spatial and temporal factors may differentially determine the influence of TGF-β on AD pathogenesis. Notably, IL-10 is also a very important pro-resolving factor. Among others, Wang *et al*. [81] demonstrated deficits in IL-10 levels in the hippocampi of AD model mice. Moreover, there is evidence of an association between a polymorphism in the IL-10 promoter and AD [82]. In the present research, we demonstrated a deficit in the IL-10 protein level in the hippocampi of KI mice at the age of 18 months and changes in the levels of this cytokine in the frontal cortex of KI mice at 12 months. Additionally, in the youngest KI offspring, the IL-10 level was negatively affected by prenatal stress. On the basis of our previous *in vitro* studies, we hypothesize that the age-dependent limitation of LXA4 and pro-resolving activation of FPR2 might influence the IL-10 level in KI mice since the LXA4 analog has been shown to increase the levels of anti-inflammatory mediators, including IL-10 [38, 56]. While it is currently difficult to reconcile the insufficient release of IL-10 in the brains of aging KI mice with cognitive disturbances, our data support the notion that pro-resolving ligand deficits may contribute to AD pathology by inhibiting RoI and inducing an imbalance of pro- and anti-inflammatory cytokines, which are additionally affected by prenatal stress.

We are aware that the present study has several limitations. The first limitation is the lack of wild-type and hAPP^NL-F/NL-F^ female mice. Likely, sex differences in AD severity, neuropathology, or brain immune response might influence or produce different results than those presented in this work. Moreover, hormonal status can also cause neurodegenerative changes related to the activation of inflammatory pathways. PS could also affect susceptibility to stress in the prenatal period and age-dependent neurodegenerative changes in both WT and KI mice. It should be noted that breeding mice is troublesome and expensive, which partly explains the limited research strategy adopted in this work. Another limitation of our research could be the lack of functional studies on the causal mechanisms. However, elucidation of the exact relationships between cognitive and behavioral deficits, RoI, and immune aging in KI mice will need better pharmacological and molecular tools than those that are presently at our disposal. Finally, it should be mentioned that night-time processes in the dampening and resetting brain systems in preparation for the coming day might be affected by aging and prenatal stress. Anderson *et al*. [83] highlighted an important role for the 10-fold decrease in pineal melatonin at night, coupled with the suppression of gut microbiome-derived butyrate, which dysregulates the wider cortisol system over the course of rising cortisol levels at night and during the cortisol awakening response. As ANXA1 is glucocorticoid receptor-induced, such night-time dysregulation in processes of dampening and resetting will be an interesting proposal to investigate regarding some FPR2 ligands.

## CONCLUSION

A better characterization of the molecular mechanisms by which endogenous resolution of inflammation represses the chronic immune brain response will aid the development of pharmaceutical strategies for treating age-related diseases, including Alzheimer's disease. The current study revealed age-dependent cognitive memory deficits in hAPP^NL-F/NL-F^ KI male mice, accompanied by an elevated Aβ42/Aβ40 ratio and *App* expression in brain tissue. Furthermore, the study revealed disturbances in the resolution of inflammation, which are determined by the age of the animal, genetic background, and brain structure. This notion was supported by the age-dependent limitation of the availability of pro-resolving FPR2 ligands, an increase in proinflammatory microglial polarization, and an increase in Aβ-mediated inflammatory FPR2 overactivation. Moreover, the present study suggested the subtle role of RoI deficits in creating brain cell senescence, which manifests as an increase in p16 and p53 pathway activation. The deficits in the levels of cytokines with pro-resolving potential, such as IL-10 and TGF-β, as well as the simultaneous increase in the release of proinflammatory cytokines (IL-1β) by polarized immune cells in the brains of old KI mice, undoubtedly indicate a lack of homeostasis, which is largely regulated by FPR2 activation. Indeed, the elevation of the inflammatory status of the KI mouse brains was also expressed as enhanced p65/NF-κB phosphorylation and decreased A20 phosphorylation. Prenatal stress has been revealed to be a substantial factor modulating the profile of inflammatory aging. However, most of the above behavioral and biochemical alterations are aggravated in a manner strongly determined by the age of the animal and the brain structure under study. It seems advisable to follow further state-of-the-art research promoting strategies to compensate for endogenous RoI deficits in the brain by exploiting the agonist-biased properties of the FPR2 receptor, thus promoting its pro-resolving effects. Moreover, additional mechanistic studies are required to understand how the modulation of FPR2 activity represses cognitive and behavioral disturbances associated with physiological and pathological aging.

## Figures and Tables

**Table 1:** Age-dependent impact of prenatal stress on the total distance traveled find the submerged platform by wild-type (WT) and APP^NL-F/NL-F^ (KI) mice in the MWM test at 9, 12, 15, and 18 months of age.

**Group**			**Behaviour Test**						
			**Training**					**Test**	
			**Day 1**	**Day 2**	**Day 3**	**Day 4**	**Day 5**	**DT**	**LTF**
Age	9 m	WT C	11.32 ± 2.71	5.18 ± 5.08	4.17 ± 3.11	3.27 ± 3.15	1.10 ± 0.68	5.22 ± 1.28	2.10 ± 0.85
		WT PS	9.20 ± 5.00	4.79 ± 4.58	1.95 ± 2.93	3.24 ± 2.85	1.56 ± 1.47	5.83 ± 1.73	2.40 ± 1.51
		KI C	8.60 ± 5.03	5.90 ± 5.10	3.32 ± 3.83	2.20 ± 2.14	2.73 ± 2.58	5.13 ± 0.48	2.00 ± 1.10
		KI PS	8.66 ± 3.79	4.91 ± 4.16	2.01 ± 1.58	1.47 ± 0.85	1.70 ± 1.92	5.67 ± 1.22	2.52 ± 1.40
	12 m	WT C	4.95 ± 2.89	5.27 ± 4.36	3.02 ± 3.23	1.69 ± 1.16	1.72 ± 2.87	5.04 ± 0.93	6.15 ± 3.53
		WT PS	6.82 ± 5.33	4.51 ± 3.90	2.39 ± 2.20	1.74 ± 1.38	1.56 ± 1.31	5.37 ± 1.10	4.44 ± 3.61
		KI C	4.15 ± 4.00	4.35 ± 3.12	2.31 ± 2.10	1.75 ± 2.19	1.52 ± 1.35	5.58 ± 071	7.26 ± 2.47
		KI PS	4.22 ± 3.96	2.38 ± 2.01	2.99 ± 3.14	2.39 ± 1.98	1.17 ± 0.70	5.22 ± 0.58	5.77 ± 4.04
	15 m	WT C	4.28 ± 3.13	3.47 ± 2.99	3.52 ± 2.89	1.18 ± 0.78	1.62 ± 1.64	5.09 ± 0.32	2.17 ± 0.23
		WT PS	5.48 ± 3.21	2.91 ± 2.38	1.74 ± 0.88	3.00 ± 2.84	2.08 ± 1.61	5.82 ± 0.60	1.76 ± 0.38
		KI C	5.23 ± 3.25	4.30 ± 2.95	1.51 ± 1.11	2.31 ± 2.77	2.22 ± 1.57	4.08 ± 0.66*	3.40 ± 2.20
		KI PS	3.45 ± 3.03	2.73 ± 2.30	1.43 ± 0.80	1.13 ± 1.18	1.77 ± 1.24	**4.86 ± 0.11^$^**	2.80 ± 2.24
	18 m	WT C	3.35 ± 1.83	1.53 ± 0.62	1.85 ± 0.92	0.86 ± 0.53	1.19 ± 0.45	6.55 ± 0.34	0.60 ± 0.28
		WT PS	5.40 ± 3.45	**3.71 ± 2.24^^^**	1.87 ± 1.36	1.38 ± 1.36	1.54 ± 1.49	**5.09 ± 0.30^^^**	**1.30 ± 0.14^^^**
		KI C	4.44 ± 2.75	2.05 ± 1.10	3.60 ± 2.63	1.18 ± 0.79	3.20 ± 1.09*	4.06 ± 0.25*	2.37 ± 0.32*
		KI PS	6.83 ± 2.49	**4.51 ± 2.73^#^**	4.05 ± 2.68	**6.64 ± 1.30^#$^**	**4.16 ± 0.85^#$^**	**3.79 ± 0.40^$^**	**3.43 ± 0.29^#$^**

**Note**: The distance traveled in the NE zone, and the latency to the first entry to the platform zone was measured in the probe trial. The data are presented as the means ± SDs, n = 3-8 mice in each group. The results were statistically evaluated using multifactorial analysis of variance (ANOVA) with Duncan’s *post hoc* test to assess the differences between the treatment groups. *p* < 0.05 * WT control *vs.* KI control; ^ WT control *vs*. WT PS; ^#^KI control *vs.* KI PS; ^$^WT PS *vs*. KI PS.

**Table 2:** Age-dependent impact of prenatal stress on exploratory behavior distance moved, velocity, and mobility in the OF test of 9-, 12-, 15-, and 18-month-old wild-type (WT) and APP^NL-F/NL-F^ (KI) mice.

**Group**			**Parameter**		
			**Distance Moved (m)**	**Velocity (cm/s)**	**Mobility (%)**
Age	9 m	WT C	108.26 ± 12,40	6.02 ± 0.69	14.29 ± 1.42
		WT PS	102.05 ± 12.54	5.67 ± 0.70	13.96 ± 1.12
		KI C	91.74 ± 11.90	5.10 ± 0.66	12.84 ± 1.36
		KI PS	89.87 ± 13.80	5.00 ± 0.77	12.90 ± 1.68
	12 m	WT C	98.44 ± 3.31	5.47 ± 0.19	13.57 ± 0.22
		WT PS	**51.80 ± 37.56^$^**	**2.88 ± 2.09^$^**	7.19 ± 5.33
		KI C	70.93 ± 9.04*	3.94 ± 0.50 *	10.65 ± 1.61
		KI PS	78.18 ± 8.51	4.35 ± 0.47	11.57 ± 0.87
	15 m	WT C	**74.37 ± 6.88^^^**	**4.13 ± 0.38^^^**	**10.09 ± 0.85^^^**
		WT PS	**67.00 ± 8.97^$^**	**3.72 ± 0.50 ^$^**	9.32 ± 1.01
		KI C	77.61 ± 10.78	4.34 ± 0.60	10.42 ± 1.15
		KI PS	70.72 ± 11.72	3.93 ± 0.65	9.94 ± 1.36
	18 m	WT C	**63.35 ± 6.73^^^**	**3.52 ± 0.37^^^**	**8.96 ± 0.63^^^**
		WT PS	**65.64 ± 13.67^$^**	**3.21 ± 0.11^$^**	8.44 ± 0.09
		KI C	**50.67 ± 3.42^#^**	**2.90 ± 0.07^#^**	**8.17 ± 0.56^#^**
		KI PS	**59.99 ± 2.06^&^**	**3.34 ± 0.12^&^**	**9.15 ± 0.43^&^**

**Note**: ANOVA with Duncan’s *post hoc* test (*p < 0.05, ^&^WT control *vs.* KI control; ^^^WT control *vs*. WT PS; ^#^KI control *vs.* KI PS; ^$^WT PS *vs*. KI PS. n = 3-4 mice in each group, error bars are standard deviations).

## Data Availability

The data and supportive information are available within the article.

## LIST OF ABBREVIATIONS

AD: Alzheimer's Disease
NFTs: Neurofibrillary Tangles
Aβ: Amyloid Beta
SPMs: Specialized Pro-resolving Mediators
PS: Prenatal Stress
