# Supplementary material for: Prenatal Stress Increases the Risk of the FPR2-related Dysfunction in the Brain's Resolution of Inflammation: A Study on the Humanized APPNL-F/NL-F Mouse Model of Alzheimer's Disease
Source: Curr Neuropharmacol. 2025 Mar 12;23(12):1600–20. doi: 10.2174/011570159X345385241004060055 (PMC12645130; doi:10.2174/011570159X345385241004060055)
Supplement: Supplementary file 1 [file CN-23-12-1600_SD1.pdf]

Supplementary Material

Prenatal Stress Increases the Risk of the FPR2-related Dysfunction in the Brain's Resolution of Inflammation: A Study on the Humanized APP$^{NL-F/NL-F}$ Mouse Model of Alzheimer's Disease

Ewa Trojan[1,*], Jakub Frydrych[1], Władysław Lasoń[1] and Agnieszka Basta-Kaim[1,*]

[1]Laboratory of Immunoendocrinology, Department of Experimental Neuroendocrinology, Maj Institute of Pharmacology, Polish Academy of Sciences, 12 Smętna St. 31-343 Kraków, Poland

The content of the supplement:

- Table 1: $F_{(df,df)}$ and *p* values in the MWM test.
- Table 2: $F_{(df,df)}$ and *p* values in the Open Field test.
- Table 3: $F_{(df,df)}$ and *p* values of *iNos, Cd40, Ym1, Arg1* mRNA expression
- Table 4: $F_{(df,df)}$ and *p* values of NLRP3 inflammasome components (NLRP3, Casp-1, ASC), A20 and the phosphorylated form of NF-κB.
- Table 5: $F_{(df,df)}$ and *p* values of FPR2, LXA4, AnxA1, p53, p16, and pRb.
- Table 6: $F_{(df,df)}$ and *p* values of Aβ42/Aβ40 ratio, and cytokines (IL-1β, IL-10, TGF-β).

1875-6190/25 $65.00+.00 **© 2025 Bentham Science Publishers**

Behaviour Test	Factor and interactions	AGE							
		9m		12m		15m		18m	
		$F_{(1.80)}$	p	$F_{(1.76)}$	p	$F_{(1.72)}$	p	$F_{(1.36)}$	p
Training									
Day1	GEN	2.27	0.135	3.02	0.085	0.53	0.468	1.89	0.176
	PS	0.91	0.352	0.97	0.327	0.15	0.696	8.89	**0.005**
	GENxPS	1.01	0.316	0.84	0.359	3.99	**0.049**	1.35	0.251
Day2	GEN	0.13	0.712	3.99	**0.049**	0.26	0.608	1.12	0.295
	PS	0.37	0.545	3.23	0.075	2.84	0.096	14.06	**0.000**
	GENxPS	0.07	0.791	0.62	0.430	0.64	0.426	0.04	0.825
Day3	GEN	0.29	0.585	0.00	0.927	11.80	**0.000**	8.86	**0.005**
	PS	3.10	0.075	0.00	0.967	7.50	**0.007**	0.12	0.729
	GENxPS	0.40	0.524	1.13	0.289	6.32	**0.014**	0.10	0.742
Day4	GEN	3.24	0.073	0.72	0.397	0.48	0.487	54.72	**<0.001**
	PS	0.45	0.504	0.65	0.421	0.38	0.538	62.72	**<0.001**
	GENxPS	0.38	0.539	0.48	0.490	8.10	**0.005**	42.82	**<0.001**
Day5	GEN	3.11	0.081	0.67	0.412	0.17	0.680	42.29	**<0.001**
	PS	0.23	0.626	0.48	0.487	0.00	0.994	3.33	0.076
	GENxPS	2.62	0.109	0.07	0.789	1.59	0.211	0.72	0.399
Test		$F_{(1.23)}$	p	$F_{(1.25)}$	p	$F_{(1.24)}$	p	$F_{(1.23)}$	p
DT	GEN	0.04	0.833	0.34	0.561	64.68	**<0.001**	80.60	**<0.001**
	PS	0.99	0.331	0.00	0.958	0.06	0.794	16.81	**0.009**
	GENxPS	0.00	0.949	1.08	0.307	0.18	0.672	8.09	**0.036**
LFE	GEN	0.00	0.967	0.73	0.402	2.35	0.138	115.68	**<0.001**
	PS	0.38	0.544	1.26	0.273	0.46	0.504	23.73	**0.002**
	GENxPS	0.54	0.818	0.00	0.939	0.01	0.895	1.07	0.350

Supplementary Table 1. Results of a two-way ANOVA investigating the effects of genotype (GEN), prenatal treatment (PS), and their interactions on the spatial learning ability of 9-, 12-, 15, and 18-month-old WT and in $APP^{NL\text{-}F/NL\text{-}F}$ knock-in mice in the MWM test. The mouse distance needed to find the submerged platform was measured over the 5-day learning trials. The distance travelled in the NE zone (DT) and the latency to the first entry to the platform zone (LFE) was measured in the probe trial. Statistically significant effects are given in bold.

Behaviour Test	Factor and interactions	$F_{(1,45)}$	p
Distance moved	GEN	1.68	0.201
	PS	2.48	0.121
	Y	17.59	**<0.001**
	GENxPS	4.28	**0.049**
	GENxY	1.33	0.274
	PSxY	1.43	0.245
	GENxPSxY	2.49	0.072
Velocity	GEN	0.94	0.336
	PS	3.51	0.067
	Y	18.32	**<0.001**
	GENxPS	5.02	**0.030**
	GENxY	1.32	0.277
	PSxY	1.07	0.370
	GENxPSxY	2.52	0.069
Mobility	GEN	0.00	0.927
	PS	2.66	0.109
	Y	18.67	**<0.001**
	GENxPS	5.58	**0.022**
	GENxY	1.06	0.371
	PSxY	1.62	0.197
	GENxPSxY	2.80	0.051

Supplementary Table 2. Results of a three-way ANOVA investigating the effects of genotype (GEN), prenatal treatment (PS), age (Y), and their interactions on the distance moved [m], mobility [%], as well as velocity [cm/s] measured in the open field test in 9-, 12-, 15, and 18-month-old WT and in $APP^{NL-F/NL-F}$ knock-in mice. Statistically significant effects are given in bold.

Gene	Factor and interactions	AGE											
		12m				15m				18m			
		Hp		Cx		Hp		Cx		Hp		Cx	
		$F_{(1,14)}$	p	$F_{(1,14)}$	p	$F_{(1,14)}$	p	$F_{(1,14)}$	p	$F_{(1,14)}$	p	$F_{(1,14)}$	p
iNos	GEN	45.20	**<0.001**	48.85	**<0.001**	67.60	**<0.001**	11.85	**0.003**	18.32	**<0.001**	14.55	**0.002**
	PS	49.04	**<0.001**	11.31	**0.004**	42.19	**<0.001**	0.22	0.643	25.06	**<0.001**	2.81	0.121
	GENxPS	0.00	0.988	1.62	0.223	6.98	**0.020**	5.17	**0.039**	0.66	0.429	3.48	0.088
Cd40	GEN	10.85	**0.006**	2.35	0.147	12.80	**0.003**	7.63	**0.017**	21.89	**<0.001**	41.52	**0.000**
	PS	7.14	**0.020**	12.88	**0.002**	0.72	0.409	7.95	**0.015**	9.29	**0.008**	72.22	**<0.001**
	GENxPS	11.29	**0.005**	0.01	0.918	2.28	0.154	2.70	0.126	1.40	0.254	9.52	**0.014**
Ym1	GEN	227.73	**<0.001**	18.52	**0.003**	5.32	**0.046**	7.04	**0.020**	8.07	**0.021**	12.61	**0.007**
	PS	178.54	**<0.001**	1.16	0.316	22.67	**0.001**	3.66	0.079	0.97	0.351	4.31	0.071
	GENxPS	157.27	**<0.001**	13.52	**0.007**	6.10	**0.035**	0.92	0.342	1.70	0.228	0.54	0.482
Arg1	GEN	44.91	**<0.001**	34.28	**<0.001**	10.23	**0.007**	6.03	**0.036**	27.85	**<0.001**	2.26	0.176
	PS	1.83	0.195	0.00	0.995	5.24	**0.040**	8.34	**0.017**	4.89	**0.042**	2.85	0.134
	GENxPS	6.45	**0.022**	0.06	0.796	4.64	0.052	1.60	0.237	**0.16**	0.694	20.66	**0.002**

Supplementary Table 3. Results of a two-way ANOVA investigating the effects of genotype (GEN), prenatal treatment (PS), and their interactions on the mRNA expression of examined proteins in the hippocampus (Hp) and Frontal cortex (Cx) in normal ageing (WT) and in $APP^{NL-F/NL-F}$ knock-in mice. Statistically significant effects are given in bold. All data was obtained from the qRT-PCR method.

Protein	Factor and interactions	Hp		Cx	
		$F_{(1,32)}$	p	$F_{(1,32)}$	p
Casp-1	GEN	44.73	**<0.001**	154.56	**<0.001**
	PS	0.80	0.375	12.27	**0.001**
	Y	13.82	**<0.001**	4.72	**0.015**
	GENxPS	0.06	0.802	1.96	0.169
	GENxY	1.92	0.162	36.82	**<0.001**
	PSxY	0.16	0.851	0.13	0.870
	GENxPSxY	0.81	0.450	11.37	**0.000**
NLRP3	GEN	79.65	**<0.001**	186.74	**<0.001**
	PS	9.62	**0.003**	9.61	**0.003**
	Y	57.02	**<0.001**	25.35	**<0.001**
	GENxPS	0.52	0.475	1.79	0.188
	GENxY	28.79	**<0.001**	44.85	**<0.001**
	PSxY	11.12	**0.000**	5.30	**0.009**
	GENxPSxY	2.07	0.141	2.68	0.081
ASC	GEN	2.80	0.103	3.93	0.054
	PS	0.02	0.174	0.01	0.916
	Y	11.13	**0.000**	1.85	0.171
	GENxPS	2.29	0.140	0.01	0.904
	GENxY	1.12	0.375	3.16	0.054
	PSxY	0.35	0.705	0.95	0.394
	GENxPSxY	1.19	0.315	0.11	0.893
A20	GEN	154.87	**<0.001**	150.49	**<0.001**
	PS	1.84	0.184	6.39	**0.016**
	Y	45.90	**<0.001**	83.76	**<0.001**
	GENxPS	0.00	0.954	0.00	1.000
	GENxY	9.33	**<0.001**	5.80	**0.006**
	PSxY	2.03	0.146	1.69	0.197
	GENxPSxY	1.77	0.185	2.65	0.084
NF-κB	GEN	56.21	**<0.001**	131.87	**<0.001**
	PS	2.64	0.114	7.28	**0.010**
	Y	4.04	**0.027**	14.36	**<0.001**
	GENxPS	0.01	0.901	0.29	0.588
	GENxY	8.77	**0.000**	3.44	**0.043**
	PSxY	0.69	0.506	2.45	0.101
	GENxPSxY	0.02	0.972	0.21	0.804

Supplementary Table 4. Results of a three-way ANOVA investigating the effects of genotype (GEN), prenatal treatment (PS), age (Y), and their interactions on the components of the NLRP3 inflammasome (NLRP3, Casp-1, ASC), A20 and the phosphorylation level of the p65 NF-κB subunit levels in the hippocampus (Hp) and Frontal cortex (Cx) in normal aging (WT) and in $APP^{NL\text{-}F/NL\text{-}F}$ knock-in mice. Statistically significant effects are given in bold. All data were obtained from ELISA.

Protein	Factor and interactions	Hp		Cx	
		$F_{(1,36)}$	*p*	$F_{(1,36)}$	*p*
FPR2	GEN	284.59	**<0.001**	248.44	**<0.001**
	PS	0.43	0.513	15.35	**0.000**
	Y	53.27	**<0.001**	56.68	**<0.001**
	GENxPS	6.24	**0.017**	20.27	**<0.001**
	GENxY	34.53	**<0.001**	114.50	**<0.001**
	PSxY	7.85	**0.001**	11.84	**<0.001**
	GENxPSxY	3.01	0.061	9.68	**<0.001**
LXA4	GEN	96.87	**<0.001**	84.61	**<0.001**
	PS	0.03	0.857	7.13	**0.011**
	Y	17.78	**<0.001**	49.77	**<0.001**
	GENxPS	1.39	0.246	0.46	0.498
	GENxY	1.53	0.229	11.34	**<0.001**
	PSxY	1.19	0.313	3.59	**0.039**
	GENxPSxY	0.23	0.790	6.21	**0.005**
AnxA1	GEN	12.64	**0.001**	54.04	**<0.001**
	PS	0.39	0.535	0.38	0.537
	Y	5.31	**0.009**	15.00	**<0.001**
	GENxPS	0.19	0.659	0.64	0.427
	GENxY	13.26	**<0.001**	6.35	**0.004**
	PSxY	2.54	0.092	1.91	0.163
	GENxPSxY	0.53	0.590	0.95	0.394
p53	GEN	31.99	**<0.001**	8.75	**0.005**
	PS	3.79	0.059	0.50	0.483
	Y	4.20	**0.023**	14.08	**<0.001**
	GENxPS	5.09	**0.030**	3.06	0.090
	GENxY	21.02	**<0.001**	7.93	**0.002**
	PSxY	2.47	0.099	0.68	0.513
	GENxPSxY	3.15	0.055	0.93	0.401
p16	GEN	142.50	**<0.001**	30.85	**<0.001**
	PS	1.42	0.241	0.61	0.439
	Y	84.29	**<0.001**	7.07	**0.002**
	GENxPS	2.95	0.094	0.00	0.978
	GENxY	29.54	**<0.001**	12.80	**<0.001**
	PSxY	1.24	0.299	7.26	**0.002**
	GENxPSxY	1.8	0.179	33.79	**<0.001**
pRb	GEN	5.38	**0.026**	34.36	**<0.001**
	PS	0.89	0.350	0.00	0.952
	Y	71.32	**<0.001**	11.84	**0.000**
	GENxPS	5.58	**0.024**	1.13	0.294
	GENxY	0.07	0.926	4.22	**0.023**
	PSxY	4.17	**0.024**	3.06	0.061
	GENxPSxY	3.08	0.059	2.91	0.069

Supplementary Table 5. Results of a three-way ANOVA investigating the effects of genotype (GEN), prenatal treatment (PS), age (Y), and their interactions on the Formyl Peptide Receptor 2 (FPR2) and its ligands (lipoksin A4 - LXA4, annexin A1 - AnxA1) and on the senescence-associated proteins: p53, p16, and pRb levels in the hippocampus (Hp) and Frontal cortex (Cx) in normal ageing (WT) and in $APP^{NL\text{-}F/NL\text{-}F}$ knock-in mice. Statistically significant effects are given in bold. All data were obtained from ELISA.

Protein	Factor and interactions	Hp		Cx	
		$F_{(1,32)}$	*p*	$F_{(1,32)}$	*p*
Aβ42/Aβ40	GEN	761.98	**<0.001**	397.82	**<0.001**
	PS	1.17	0.283	1.92	0.176
	Y	1.69	0.192	0.57	0.567
	GENxPS	1.77	0.187	4.28	**0.042**
	GENxY	1.40	0.254	1.58	0.213
	PSxY	6.32	**0.002**	4.16	**0.020**
	GENxPSxY	5.32	**0.007**	7.92	**0.000**
IL-1β	GEN	163.22	**<0.001**	28.24	**<0.001**
	PS	5.80	**0.021**	0.00	0.987
	Y	0.21	0.805	11.41	**0.000**
	GENxPS	6.16	**0.018**	0.24	0.624
	GENxY	2.40	0.106	20.63	**<0.001**
	PSxY	1.24	0.302	0.10	0.905
	GENxPSxY	1.66	0.205	0.26	0.766
IL-10	GEN	5.71	**0.023**	0.18	0.892
	PS	7.09	**0.012**	5.02	**0.031**
	Y	5.54	**0.008**	0.11	0.894
	GENxPS	0.47	0.494	21.55	**<0.001**
	GENxY	8.17	**0.001**	9.67	**<0.001**
	PSxY	12.37	**0.000**	1.39	0.262
	GENxPSxY	0.06	0.933	2.54	0.092
TGF-β	GEN	16.20	**0.000**	12.44	**0.001**
	PS	0.18	0.668	3.17	0.083
	Y	2.27	0.119	31.39	**<0.001**
	GENxPS	3.59	0.066	2.44	0.127
	GENxY	0.94	0.400	1.58	0.219
	PSxY	1.73	0.192	4.27	**0.021**
	GENxPSxY	0.87	0.426	10.75	**0.000**

Supplementary Table 6. Results of a three-way ANOVA investigating the effects of genotype (GEN), prenatal treatment (PS), age (Y), and their interactions on the components of the Aβ42/Aβ40 ratio, and on the pro- (IL-1β) and anti-inflammatory cytokines (IL-10, TGF-β) levels in the hippocampus (Hp) and Frontal cortex (Cx) in normal aging (WT) and $APP^{NL-F/NL-F}$ knock-in mice. Statistically significant effects are given in bold. Data for Aβ42/Aβ40, IL-1β, IL-10, and TGF-β were obtained from ELISA test.
